# Fibroblast Growth Factor 21 Promotes Vascular Smooth Muscle Cell Contractile Polarization via p38 Mitogen-Activated Protein Kinase-Promoted Serum Response Factor Phosphorylation

**DOI:** 10.34133/research.0815

**Published:** 2025-08-05

**Authors:** Mengmeng Zhu, Wenya Zhu, Jianyuan Pan, Ziyi Chen, Yali Yan, Shengnan Wang, Tingting Zhang, Xiaoxiao Yang, Hongmei Xu, Xiangyong Kong, Hao Hu, Suowen Xu, Xia Zhang, Buchun Zhang, Chenzhong Liao, Yajun Duan, Shu Yang, Yuanli Chen

**Affiliations:** ^1^ Key Laboratory of Metabolism and Regulation for Major Diseases of Anhui Higher Education Institutes, Anhui Provincial International Science and Technology Cooperation Base for Major Metabolic Diseases and Nutritional Interventions, School of Food and Biological Engineering, Hefei University of Technology, Hefei, China.; ^2^ Department of Cardiology, The First Affiliated Hospital of USTC, Division of Life Sciences and Medicine, University of Science and Technology of China, Hefei, China.; ^3^ Department of Endocrinology, The First Affiliated Hospital of USTC, Division of Life Sciences and Medicine, University of Science and Technology of China, Hefei, China.; ^4^ Tianjin Baodi Hospital, Baodi Clinical College of Tianjin Medical University, Tianjin, China.; ^5^ Department of Geriatrics, Peking University Shenzhen Hospital, Shenzhen, Guangdong 518036, China.; ^6^ Department of Geriatrics, The First Affiliated Hospital of Southern University of Science and Technology (Shenzhen People’s Hospital), Shenzhen, Guangdong 518020, China.

## Abstract

Phenotypic abnormalities in vascular smooth muscle cells (VSMCs) are believed to play essential roles in the progression of vascular diseases. Here, we explored the impact of fibroblast growth factor 21 (FGF21) on the phenotypic transition of VSMCs. Our findings revealed that FGF21 expression was substantially down-regulated in both human and mouse neointimal regions. Additionally, plasma FGF21 levels were lower in patients with atherosclerotic coronary artery disease (ASCAD) compared to those without ASCAD. Similarly, patients with restenosis exhibited reduced FGF21 levels compared to those without restenosis. In vivo, FGF21 deficiency accelerated intimal hyperplasia and decreased the number of contractile VSMCs in mouse neointima. However, hepatocyte-specific FGF21 knockout had no effect on ligation-induced intimal hyperplasia. Conversely, administration of recombinant FGF21 protein reduced neointima formation. This effect was abolished in mice with β-klotho VSMC-specific knockout, suggesting a direct effect of FGF21 on VSMCs. In vitro, FGF21 could promote the contractile phenotype transition of human aortic smooth muscle cells under basal or platelet-derived growth factor-BB incubation conditions. Furthermore, FGF21 activation led to the phosphorylation of p38 mitogen-activated protein kinase (p38 MAPK), which subsequently formed a complex with the serum response factor (SRF)–myocardin complex. This complex increased the phosphorylation of SRF at serine 224, thereby enhancing the transcription activation of the SRF–myocardin complex. Finally, we revealed that treatment with the FGF21 analog efruxifermin or activation of p38 MAPK using anisomycin effectively inhibited neointima formation. Taken together, these results indicate that modulating FGF21 or its subsequent signal pathways could serve as a therapeutic strategy for vascular diseases characterized by abnormal VSMC phenotypic transition.

## Introduction

Cardiovascular diseases, which are characterized by the highest morbidity and mortality rates, pose a significant threat to human health [1–3]. These diseases are often manifestations of systemic vascular pathology in the heart and surrounding blood vessels [4]. Phenotypic changes in vascular smooth muscle cells (VSMCs) are considered to underlie several human vascular diseases, including hypertension, atherosclerosis, in-stent restenosis (ISR), and graft artery disease [5–8]. In a healthy vascular system, VSMCs can maintain the stability of vascular tension and the internal environment of the vasculature by expressing a series of contractile proteins [9]. However, following vascular injury, under the influence of circulating factors, growth factors, and circular RNAs, VSMCs undergo a series of changes, including increased proliferation, secretion of extracellular matrix-degrading enzymes, and decreased contractility [10,11]. This phenotypic transition drives VSMCs to migrate toward the vascular intima, initiating an adaptive reorganization process. It may eventually exacerbate the occurrence of intimal hyperplasia and restenosis [12].

The fibroblast growth factor (FGF) family consists of 22 members and encompasses a broad spectrum of biological functions, including cell growth, development, angiogenesis, and wound healing [13–16]. FGF family genes have been reported to regulate the plasticity of VSMCs. For instance, FGF1, FGF2, and FGF23 have been shown to stimulate VSMC proliferation and intimal hyperplasia formation following vascular injury [17–19]. In contrast, FGF12 has been demonstrated to induce the quiescent and contractile phenotype in VSMCs and inhibit neointima formation in injured vessels via the p38 mitogen-activated protein kinase (p38 MAPK) pathway [20]. Fibroblast growth factor 21 (FGF21) is primarily expressed in the liver and acts on tissues such as the liver, adipose tissue, and pancreas to regulate carbohydrate metabolism [21,22]. FGF21 has been identified as a stress response factor. Serum FGF21 is elevated in newly diagnosed type 2 diabetes patients and is positively correlated with carotid and iliac lesions in patients with subclinical atherosclerosis [23]. Interestingly, serum FGF21 levels are positively correlated with carotid intima–media thickness in women but not in men [24]. Previous studies have highlighted the vasoprotective properties of FGF21, which are independent of its effects on blood glucose control and insulin sensitivity [25–27]. For example, by binding to its receptors, β-klotho (KLB) and FGF receptor 1 (FGFR1), FGF21 down-regulates hepatic sterol regulatory element binding protein-2 to inhibit cholesterol biosynthesis and induce adiponectin to inhibit atherosclerosis [28]. FGF21 may also alleviate endothelial dysfunction and macrophage foam cell formation through multiple pathways [29]. Additionally, FGF21 inhibits VSMC migration and proliferation by activating the angiotensin-converting enzyme 2 (ACE2)/angiotensin-(1-7) axis and inhibiting the FGFR1–spleen tyrosine kinase–NLRP3 inflammasome pathway [27,30]. Despite these findings, the direct effects of FGF21 on VSMCs and the detailed underlying mechanisms remain incompletely understood.

Here, we initially determined that FGF21 expression was substantially reduced in the neointimal area of human lesions from patients with carotid artery stenosis. Furthermore, serum FGF21 levels were lower in atherosclerotic coronary artery disease (ASCAD) patients compared to those in non-ASCAD controls. Similarly, FGF21 levels were reduced in restenosis patients compared with FGF21 levels in those without restenosis. Using mouse models of carotid artery ligation and femoral artery guidewire injury, we revealed that FGF21 deficiency accelerated neointima formation by promoting the phenotypic transition of VSMCs to a proliferative phenotype. We also elucidated the underlying mechanisms by which FGF21 affected VSMC phenotype transition. Specifically, FGF21 acts through the p38 MAPK pathway to induce serum response factor (SRF) phosphorylation, thereby enhancing the activation of the SRF/myocardin (MYOCD) complex.

## Results

### FGF21 expression is significantly down-regulated in intimal hyperplasia

**Fig. 1.**
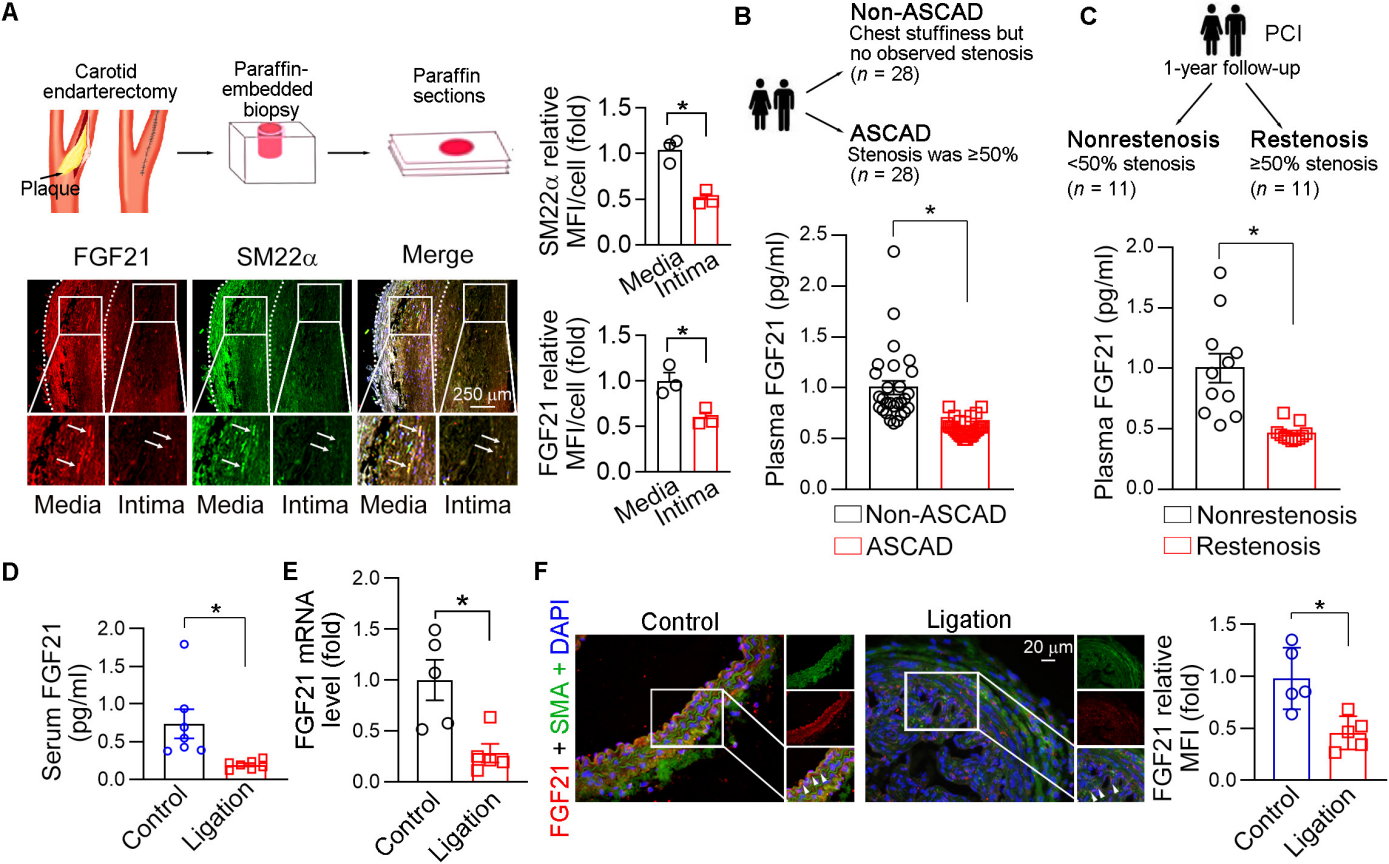
Fibroblast growth factor 21 (FGF21) expression was significantly down-regulated in neointimal hyperplasia. (A) Paraffin sections of the carotid artery were prepared and used to determine the expression of FGF21 and smooth muscle 22α (SM22α) by immunofluorescence staining (*n* = 3). Bars: 250 μm. (B) Plasma FGF21 levels were determined by enzyme-linked immunosorbent assay (ELISA) in atherosclerotic coronary artery disease (ASCAD) patients and non-ASCAD patients (*n* = 28). (C) FGF21 levels in the plasma of patients who underwent percutaneous coronary intervention (PCI) with and without restenosis were determined via ELISA (*n* = 11). (D to F) Carotid artery ligation was performed in wild-type (WT) mice. Serum FGF21 levels were determined by ELISA (*n* = 7) (D). FGF21 messenger RNA (mRNA) expression was determined by quantitative polymerase chain reaction (qPCR) (*n* = 5) (E). The control and ligated carotid artery cross-sections were subjected to immunofluorescence staining to determine FGF21 and smooth muscle α-actin (SMA) expression (*n* = 5) (F). Data information: Data are expressed as mean ± SD. Student *t* test, *P < 0.05. MFI, mean fluorescence intensity.

FGF21 has been shown to exert protective effects against atherosclerosis. To elucidate the role of FGF21 in intimal hyperplasia, carotid artery samples were collected from 3 patients with stenosis who underwent carotid endarterectomy. FGF21 expression was determined by immunofluorescence staining. Compared to that in the medial layers, FGF21 expression was markedly lower in the neointimal areas, which was accompanied by reduced smooth muscle 22α (SM22α) expression in the neointimal areas (Fig. 1A). To further determine whether FGF21 is involved in proliferative and obstructive vascular diseases, plasma samples were collected from patients with ASCAD and non-ASCAD controls, as well as from patients who underwent percutaneous coronary intervention (PCI) with or without restenosis. The baseline characteristics of all participants are listed in Table S2. Serum FGF21 levels were substantially lower in ASCAD patients compared to those in non-ASCAD patients (*P* < 0.0001, *n* = 28; Fig. 1B). Additionally, serum FGF21 levels were markedly lower in patients with ISR compared to those without restenosis during the 1-year follow-up, as shown by enzyme-linked immunosorbent assay (ELISA; *P* < 0.0001, *n* = 11; Fig. 1C).

Subsequently, we established an intimal hyperplasia model in C57BL/6J mice through carotid artery ligation surgery. We observed that FGF21 levels were decreased in the serum (Fig. 1D) and aorta (Fig. 1E) of the model group compared to those in the control group. Consistent results were obtained from immunofluorescence staining of carotid artery sections that FGF21 in smooth muscle α-actin (SMA)-positive cells was reduced in the aorta of the model group (Fig. 1F). In summary, the above results suggest that FGF21 was reduced during the process of intimal hyperplasia.

### FGF21 inhibits intimal hyperplasia by acting directly on the aorta

**Fig. 2.**
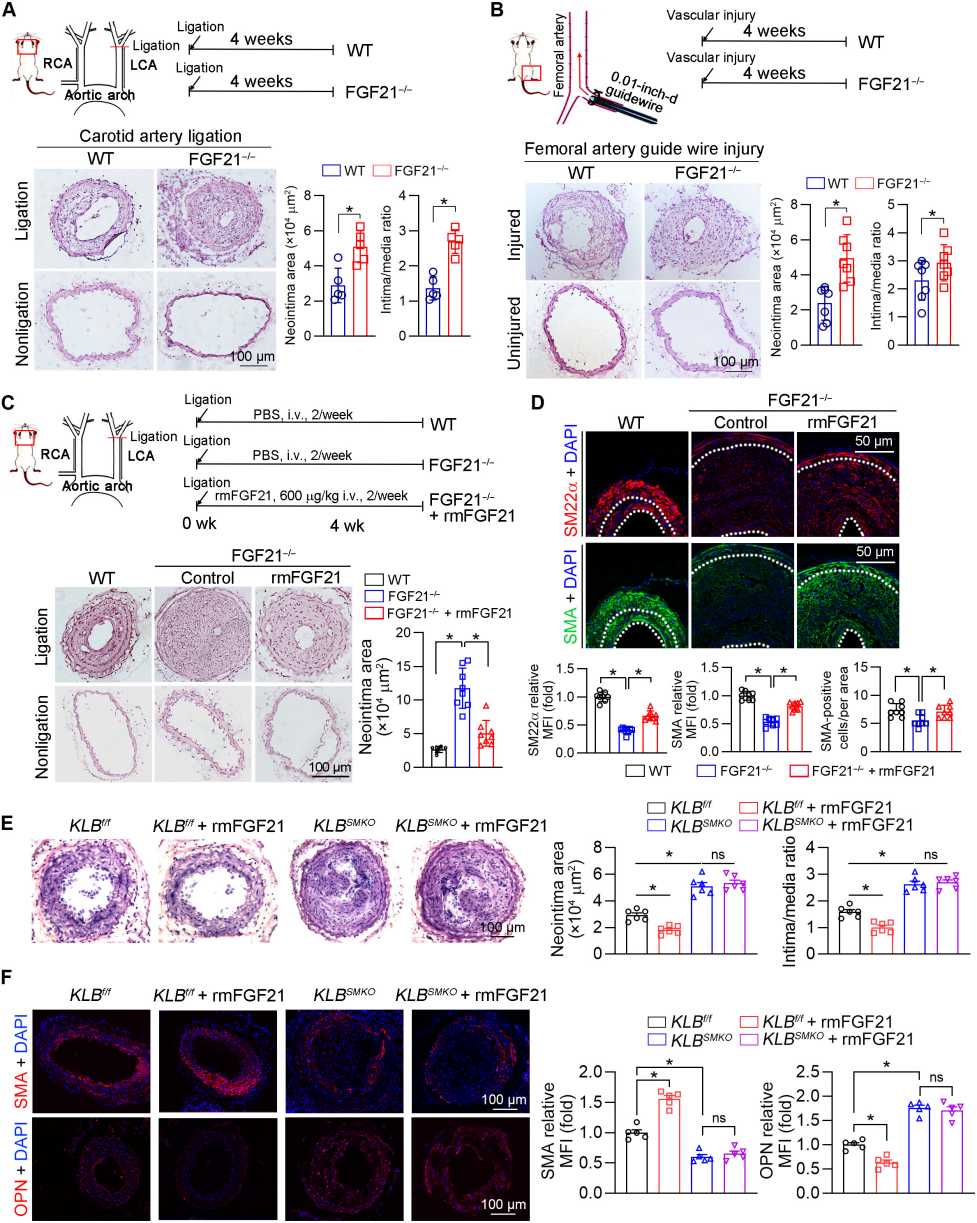
FGF21 inhibits neointimal formation by binding to its receptor in the aorta. (A and B) Carotid artery ligation (*n* = 5) and femoral artery guidewire injury (*n* = 7) were performed in WT and FGF21^−/−^ mice. Both the left and right carotid arteries and the femoral artery were collected, and cross-sections were prepared for hematoxylin and eosin (HE) staining for morphological analysis with quantitative analysis of the neointima and media areas. Data information: Data are expressed as mean ± SD. Student *t* test, *P < 0.05. (C and D) Left carotid artery ligation was performed on female C57BL/6J and FGF21^−/−^ mice (*n* = 8), and FGF21^−/−^ mice were intravenously injected with recombinant mouse FGF21 protein (rmFGF21; 600 μg/kg) twice a week for 4 weeks. At the end of the experiment, carotid artery samples were individually collected and used for the following assay: (C) HE staining for morphological analysis with quantitative analysis of neointima and media areas. (D) The expression of SMA and SM22α in neointimal areas was determined by immunofluorescence staining. (E and F) Female KLB^f/f^ and KLB^SMKO^ mice (*n* = 6) were subjected to left carotid artery ligation, and rmFGF21 (600 μg/kg) was intravenously injected twice a week for 4 weeks. At the end of the experiment, carotid artery samples were individually collected and used for the following assay: (E) HE staining for morphological analysis with quantitative analysis of neointima and media areas. (F) The expression of SMA and osteopontin (OPN) in neointimal areas was determined by immunofluorescence staining (*n* = 5). Data information: The data are expressed as mean ± SD. Student *t* test (2 groups), 1-way analysis of variance (ANOVA) or 2-way ANOVA followed by Tukey’s test (more than 2 groups), *P < 0.05; ns, not significant. RCA, right carotid artery; LCA, left carotid artery; PBS, phosphate-buffered saline; i.v., intravenous.

To investigate the impact of FGF21 on intimal hyperplasia, we examined its effects on neointima formation induced by partial ligation or guidewire injury in the carotid and femoral arteries, respectively. In wild-type (WT) mice, compared to those in the sham operation group, severe neointima was observed in the left carotid artery or femoral artery after ligation or injury (Fig. 2A and B). Notably, neointima formation was markedly exacerbated in the FGF21 knockout (FGF21^−/−^) mice in both experimental models (Fig. 2A and B). To further investigate the role of FGF21 in intimal hyperplasia, we administered recombinant mouse FGF21 protein (rmFGF21) intravenously to FGF21^−/−^ mice subjected to carotid artery ligation. Histological analysis using hematoxylin and eosin (HE) staining revealed that rmFGF21 supplementation alleviated FGF21 knockout-induced intimal hyperplasia in mice (Fig. 2C). Additionally, the expression of contractile genes (SMA and SM22α) was increased by rmFGF21 (Fig. 2D). Moreover, rmFGF21 treatment partially restored the reduction in SMA-positive cell numbers induced by FGF21 knockout in the same size of neointima areas (Fig. 2D), implying that rmFGF21 restored the FGF21 knockout-induced reduction in the number of contractile smooth muscle cells (SMCs) in the neointimal areas.

FGF21 is supposed to be an endocrine hormone primarily secreted by the liver. Therefore, we used mice with hepatocyte-specific knockout of FGF21 (FGF21^HepKO^) [31] to determine if the protective effect on the neointima formation of FGF21 depended on its metabolic function. We established a carotid artery ligation-induced intimal hyperplasia model in these mice. HE staining results showed that hepatocyte-specific knockout of FGF21 had no effect on intimal hyperplasia in mice (Fig. S1A and B). To investigate whether FGF21 inhibits intimal hyperplasia by directly acting on the aorta, we generated VSMC-specific KLB knockout mice (KLB^f/f^SM22-Cre, KLB^SMKO^, Fig. S1C). In these mice, KLB messenger RNA expression was reduced in the aorta but remained unaffected in other tissues such as the liver, brain, kidney, or adipose tissue (Fig. S1D). We also confirmed that KLB protein was totally knocked out in primary mouse aortic SMCs (Fig. S1E to G). We then assessed the impact of KLB knockout on neointima formation using the carotid artery ligation model. Our results showed that KLB knockout accelerated ligation-induced neointima formation (Fig. 2E). Importantly, rmFGF21-reduced neointima formation was completely abolished in KLB^SMKO^ mice (Fig. 2E), indicating that KLB is essential for mediating the effects of FGF21 on intimal hyperplasia. Moreover, we examined the expression of the key markers of VSMC phenotypic switching. In WT mice, rmFGF21 treatment increased the expression of the contractile marker SMA and decreased the expression of the synthetic marker osteopontin (OPN). However, these effects were blocked in KLB^SMKO^ mice (Fig. 2F). Taken together, the above data suggest that FGF21 acts directly on a VSMC by binding to its receptor KLB, thereby regulating VSMC phenotypic switching and inhibiting intimal hyperplasia.

### FGF21 promotes VSMC contraction phenotype switching through the up-regulation of SRF and MYOCD

Phenotype switching of VSMCs is an essential process in the development of vascular occlusive diseases. We next determined the effect of FGF21 on human aortic smooth muscle cells (HASMC) phenotype switching. We first confirmed the efficacy of FGF21 overexpression and knockdown in HASMCs (Fig. S2A to F). Indeed, we determined that FGF21 overexpression diminished the proliferation and migration of HASMCs (Fig. S2G and I). Conversely, small interfering RNA (siRNA)-mediated knockdown of FGF21 expression in HASMCs led to increased cell growth and migration compared to those in controls (Fig. S2H and J).

**Fig. 3.**
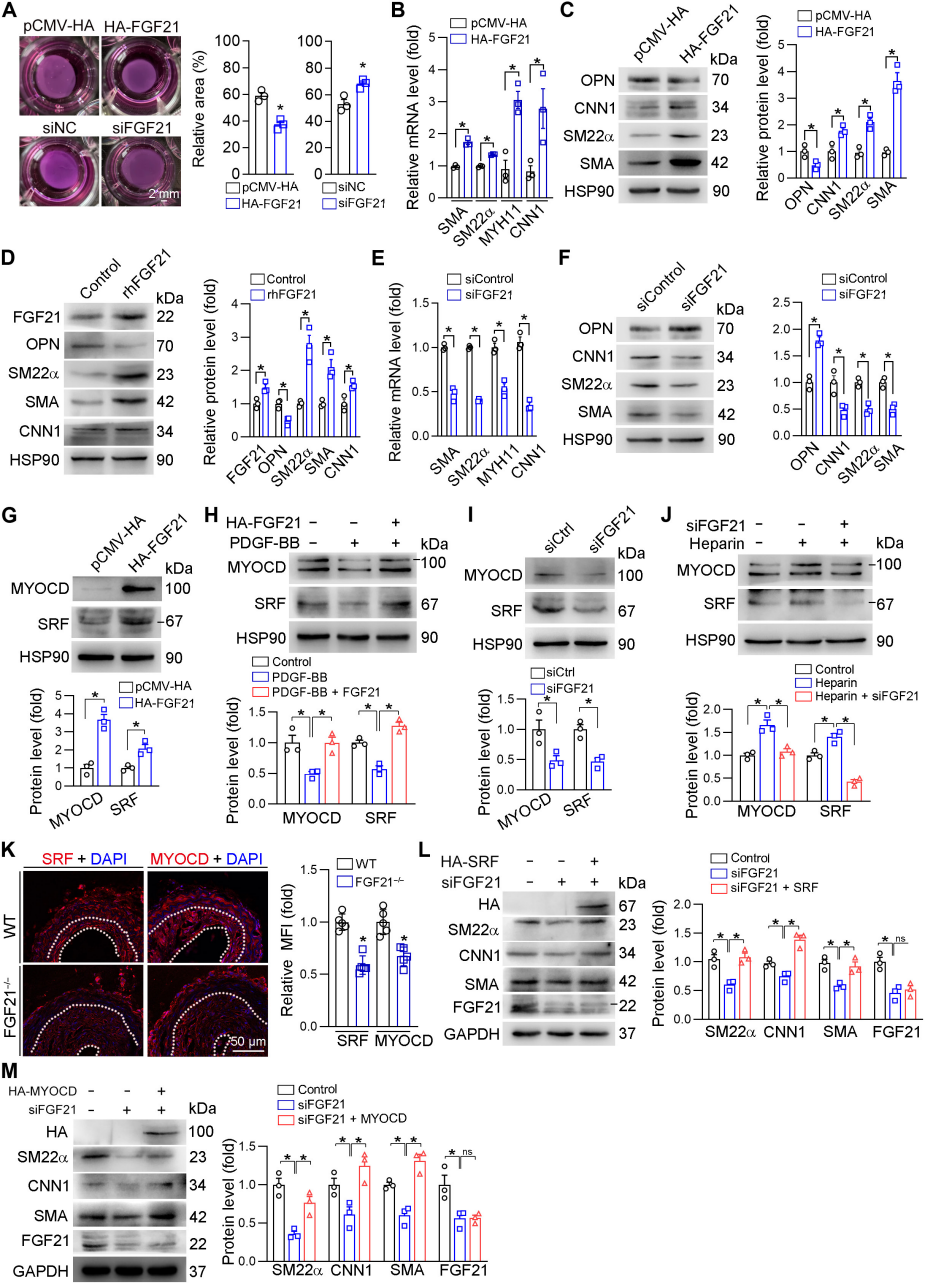
FGF21 promotes vascular smooth muscle cell (VSMC) contractile phenotype switching through up-regulation of serum response factor (SRF) and myocardin (MYOCD). Human aortic smooth muscle cells (HASMCs) were transfected with pCMV-hemagglutinin (HA)/HA-FGF21 or siCtrl/siFGF21 for 24 h in serum-free medium and cultured with complete medium for 24 h. Then, the HASMCs were harvested and mixed with collagen at a ratio of 1:4 for another 48 h, after which the size of the collagen gel was evaluated (*n* = 3) (A). FGF21, SMA, SM22α, myosin heavy chain 11 (MYH11), and calponin 1 (CNN1) mRNA levels were determined by qPCR (*n* = 3) (B and E); FGF21, SMA, CNN1, SM22α, and OPN protein expression was determined by Western blotting (*n* = 3) (C and F). (D) HASMCs were treated with recombinant human FGF21 (rhFGF21) protein (0.5 mg/ml) in serum-free medium for 24 h. The protein expression of FGF21, SM22α, SMA, and CNN1 was determined by Western blotting (*n* = 3). (G to J) HASMCs in a 6-well plate were transfected with pCMV-HA/HA-FGF21 or siCtrl/siFGF21 for 24 h. Then, the cells were treated with or without platelet-derived growth factor-BB (PDGF-BB; 40 ng/ml) or heparin (100 μg/ml) for 24 h. The expression of SRF and MYOCD was determined by Western blotting (*n* = 3). (K) The expression of SRF and MYOCD in the left carotid artery was determined by immunofluorescence staining and quantification of the MFI (*n* = 5). (L and M) HASMCs in a 6-well plate were transfected with siCtrl/siFGF21 and HA-SRF or HA-MYOCD for 24 h. The expression of HA, SM22α, CNN1, and SMA was determined by Western blotting (*n* = 3). Data information: Data are expressed as mean ± SD. Student *t* test (2 groups) or one-way ANOVA followed by Tukey’s test (more than 2 groups), *P < 0.05.

Collagen gel contraction assays revealed that overexpression of FGF21 increased the contractile activity of HASMCs (Fig. 3A). Conversely, siFGF21-transfected HASMCs exhibited reduced contractility (Fig. 3A). Furthermore, FGF21 overexpression promoted the expression of key contractile genes, including SMA, SM22α, myosin heavy chain 11 (MYH11), and calponin 1 (CNN1) (Fig. 3B and C). In contrast, the expression of OPN, a marker associated with the proliferative phenotype of VSMCs, was reduced by FGF21 overexpression (Fig. 3C). To further validate these findings, we treated HASMCs with recombinant human FGF21 (rhFGF21) protein. rhFGF21 increased the expression of SM22α, SMA, and CNN1 (Fig. 3D), which was abolished by siRNA-mediated KLB knockdown (Fig. S3A). In contrast, siRNA-mediated knockdown of FGF21 resulted in decreased messenger RNA and protein expression of contractile genes (SMA, SM22α, MYH11, and CNN1) while simultaneously increasing OPN protein expression (Fig. 3E and F). Collectively, these data suggest that FGF21 directly influences the phenotypic switching of HASMCs, promoting a contractile phenotype and inhibiting the proliferative phenotype.

Platelet-derived growth factor-BB (PDGF-BB) is a well-established mediator of VSMC phenotypic switching that promotes VSMC proliferation. We confirmed that PDGF-BB treatment inhibited the expression of contractile genes, including CNN1, SMA, and SM22α (Fig. S3B). Notably, PDGF-BB also inhibited the expression of FGF21 (Fig. S3B). More importantly, we observed that PDGF-BB-mediated inhibition of FGF21, CNN1, SMA, and SM22α expression was reversed by either FGF21 overexpression or treatment with rhFGF21, regardless of serum exposure (Fig. S3C and D). Heparin, known for its antiproliferative, anticoagulant, and antithrombin effects, has been demonstrated to maintain VSMCs in a contractile phenotype [32,33]. We determined that heparin increased the expression of FGF21, CNN1, SMA, and SM22α (Fig. S3E), which was abolished by siRNA-mediated knockdown of FGF21 (Fig. S3F).

SRF and MYOCD are the main transcription factors controlling the expression of contractile genes by forming a complex that binds to the CArG box, thereby promoting the expression of contractile proteins. We determined that FGF21 overexpression or rhFGF21 protein increased SRF and MYOCD expression (Fig. 3G and Fig. S4A and B) and also restored PDGF-BB-inhibited SRF and MYOCD expression with or without serum starvation (Fig. 3H and Fig. S4C and D). Reciprocally, FGF21 siRNA inhibited the basal or heparin-induced expression of SRF and MYOCD (Fig. 3I and J and Fig. S4E). Additionally, FGF21 overexpression increased the nuclear abundance of SRF and MYOCD (Fig. S4F and G). In vivo, compared with those in the WT group, the SRF and MYOCD expression in the neointimal area was substantially lower in the FGF21^−/−^ mice (Fig. 3K), which was restored by rmFGF21 supplementation (Fig. S5A). In addition, the SRF and MYOCD expression in the neointimal area was attenuated in KLB^SMKO^ mice compared with that in littermate controls, and FGF21-promoted SRF and MYOCD expression was blocked by KLB knockout in VSMCs (Fig. S5B). Moreover, the FGF21 siRNA-mediated inhibition of contractile genes’ expression in HASMCs was reversed by overexpression of either SRF or MYOCD (Fig. 3L and M).

### FGF21 promotes the phosphorylation of the SRF Ser224 site by p38 MAPK

**Fig. 4.**
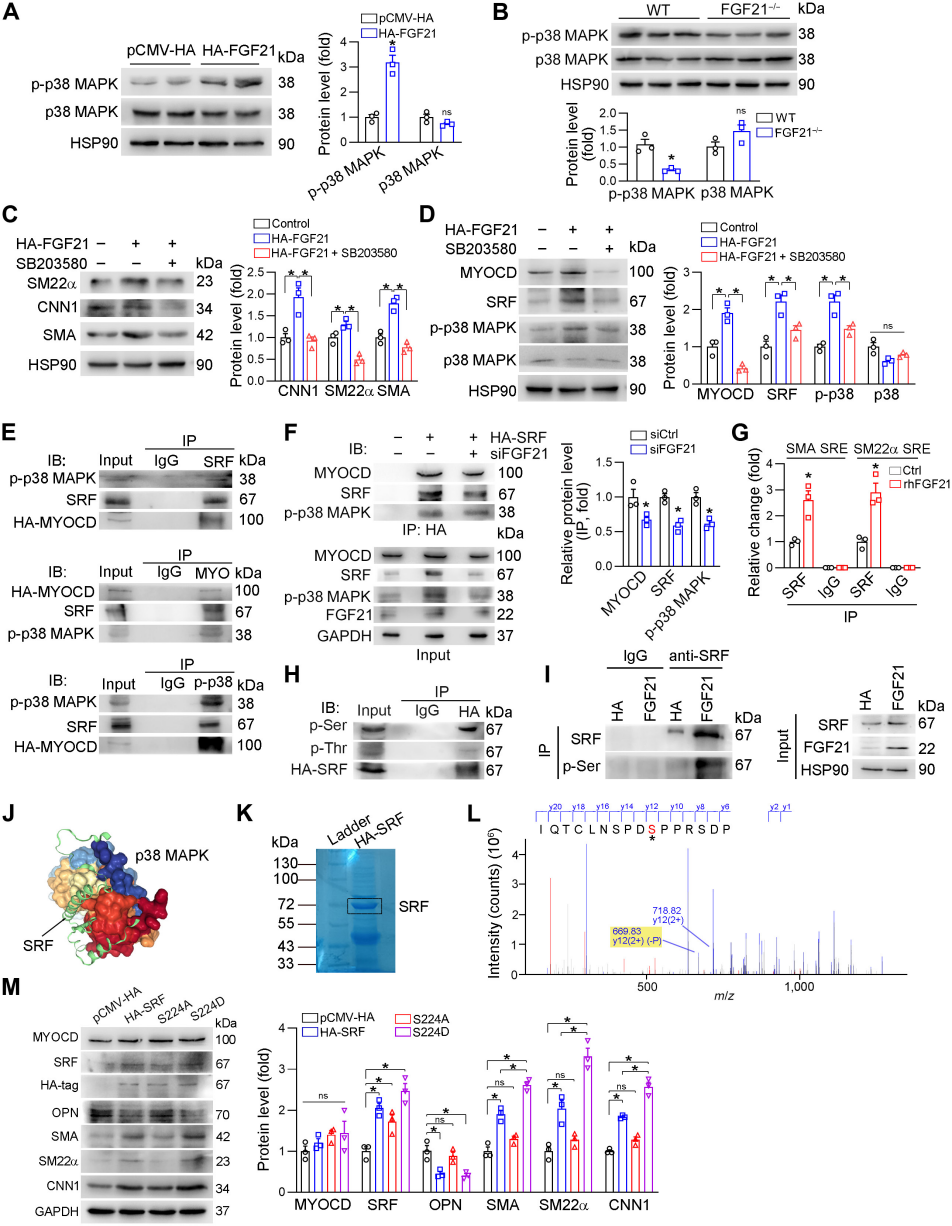
FGF21 promotes phosphorylation of the SRF Ser224 site by p38 mitogen-activated protein kinase (p38 MAPK), which in turn promotes recruitment of downstream contractile genes. (A) HASMCs in a 6-well plate were transfected with pCMV-HA/HA-FGF21 for 24 h. The expression of p38 MAPK and p-p38 MAPK was determined by Western blotting (*n* = 3). (B) The expression of p38 MAPK and p-p38 MAPK in the aortas of the WT and FGF21^−/−^ mice subjected to carotid artery ligation was determined by Western blotting (*n* = 3). (C and D) HASMCs were transfected with pCMV-HA or HA-FGF21 for 24 h in serum-free medium. Then, the cells were treated with SB203580 (10 μmol/l) for 12 h. The expression of SRF, MYOCD, p38 MAPK, p-p38 MAPK, SMA, CNN1, and SM22α was determined by Western blotting (*n* = 3). (E) HASMCs were transfected with pCMV-HA or HA-MYOCD for 24 h, after which the cells were transferred to complete medium for 24 h. The resulting cell lysates were subjected to immunoprecipitation (IP) with anti-p-p38 MAPK, anti-SRF, or anti-MYOCD antibodies. The pulled-down complexes and input cell lysates were analyzed by Western blotting with the anti-p-p38 MAPK, anti-SRF, or anti-MYOCD antibodies. (F) HASMCs were transfected with pCMV-HA, HA-SRF, or HA-SRF plus siFGF21 for 24 h in serum-free medium, after which the cells were switched to complete medium for 24 h. The IP experiment was performed with HA-tagged magnetic beads, followed by immunoblotting with anti-MYOCD, SRF, and p38 MAPK antibodies (*n* = 3). (G) Chromatin was isolated from HASMCs with or without rhFGF21 treatment (0.5 mg/ml). After determination of input, IP was conducted with normal immunoglobulin G (IgG) or SRF antibodies, followed by qPCR. *P < 0.05 versus the corresponding control (*n* = 3). (H) HASMCs were transfected with pCMV-HA or HA-SRF for 24 h in serum-free medium, after which the cells were switched to complete medium and cultured for 24 h. An IP experiment was performed with HA-tagged magnetic beads, followed by immunoblotting with anti-phospho-serine or anti-phospho-threonine antibody. (I) HASMCs were transfected with pCMV-HA or HA-FGF21 for 24 h in serum-free medium, after which the cells were switched to complete medium and cultured for 24 h. IP experiments were performed with protein A/G magnetic beads, IgG, and anti-SRF antibody, followed by immunoblotting with anti-phospho-serine and anti-SRF antibodies. (J) Molecular docking of SRF and p38 MAPK proteins. (K and L) HASMCs were transfected with HA-SRF for 24 h in serum-free medium, after which the cells were switched to complete medium supplemented with anisomycin for 24 h. The cell lysate was separated via sodium dodecyl sulfate–polyacrylamide gel electrophoresis (SDS-PAGE). The band corresponding to the molecular weight of SRF was cut and digested to carry out a liquid chromatography–tandem mass spectrometry (LC-MS/MS) assay. (M) HASMCs were transfected with HA-SRF, HA-SRF S224A, or HA-SRF S224D for 24 h in serum-free medium, after which the cells were switched to complete medium for 24 h. The protein expression of SRF, MYOCD, HA, SMA, SM22α, CNN1, and OPN was determined by Western blotting (*n* = 3). Data information: Data are expressed as mean ± SD. Student *t* test (2 groups) or one-way ANOVA followed by Tukey’s test (more than 2 groups), *P < 0.05; ns, not significant. IB, immunoblotting.

The MAPK signaling pathway is crucial for cell proliferation and differentiation, including in HASMCs [34–36]. To further define the mechanisms by which FGF21 affects VSMC phenotypic switching, we investigated its impact on the MAPK signaling pathway. We found that FGF21 overexpression had little effect on the phosphorylation of JNK or ERK1/2 (Fig. S6A). Additionally, neither p-GSK3β nor GSK3β, an enzyme known to phosphorylate SRF, was affected by FGF21 (Fig. S6B). The AMPK and AKT signaling pathways also contribute to the phenotype switching of VSMCs [37,38]. Our experimental results indicate that FGF21 had no marked effect on either the AMPK or AKT signaling pathways (Fig. S6C). However, FGF21 substantially increased the phosphorylation of p38 MAPK (p-p38 MAPK) in HASMCs, regardless of serum starvation (Fig. 4A and Fig. S7A). In vivo, the protein expression of p-p38 MAPK was reduced in the aortas of FGF21^−/−^ mice (Fig. 4B). Similarly, the expression of p-p38 MAPK in the carotid artery was reduced in KLB^SMKO^ mice compared to that in KLB^f/f^ mice (Fig. S7B). Importantly, the FGF21-promoted p-p38 MAPK expression was blocked by KLB knockout in VSMCs (Fig. S7B). To confirm the role of p38 MAPK in FGF21 signaling, we treated HASMCs with the p38 MAPK inhibitor SB203580. Indeed, SB203580 reduced the expression of FGF21-induced contractile genes (CNN1, SM22α, and SMA), as well as MYOCD, SRF, and p-p38 MAPK (Fig. 4C and D).

To further elucidate the underlying mechanism of p-p38 MAPK-induced SRF/MYOCD activity, we hypothesized that p38 MAPK may directly interact with SRF/MYOCD. Indeed, we found that p-p38 MAPK interacted with the SRF/MYOCD complex as determined by a co-immunoprecipitation assay (Fig. 4E). Furthermore, the interaction of p-p38 MAPK with the SRF/MYOCD complex was reduced by FGF21 siRNA treatment (Fig. 4F). Chromatin immunoprecipitation followed by quantitative polymerase chain reaction (ChIP-qPCR) experiments demonstrated that rhFGF21 supplementation increased the SRF recruitment to the endogenous SMC differentiation marker genes (SMA and SM22α) (Fig. 4G). p38 MAPK is a serine/threonine protein kinase. We initially predicted the potential phosphorylation site of SRF or MYOCD by p38 MAPK with Group-based Prediction System (GPS version 6.0, http://gps.biocuckoo.cn/). SRF, but not MYOCD, was predicted to be a direct substrate of p38 MAPK at serine 224 (Ser224). Indeed, we detected strong serine phosphorylation but not threonine phosphorylation in SRF protein (Fig. 4H). In addition, FGF21 overexpression increased the serine phosphorylation of SRF (Fig. 4I). Molecular docking simulations revealed that SRF Ser224 was present at the contact surface between SRF and p38 MAPK (Fig. 4J). We subsequently performed an LC-MS/MS assay to determine the phosphorylation site of SRF. We identified the sole phosphorylation site at Ser224 in SRF (Fig. 4K and L). Therefore, we generated an SRF overexpression vector with Ser224 mutations (nonphosphorylated SRF: mutation of serine 224 to alanine, S224A; constitutively phosphorylated SRF: mutation of serine 224 to aspartate, S224D). Overexpression of WT SRF or constitutively phosphorylated SRF (S224D) increased SMA, SM22α, and CNN1 expression but inhibited OPN expression (Fig. 4M). However, the expression of nonphosphorylated SRF (S224A) had little effect on the expression of these genes (Fig. 4M). Taken together, the data in Fig. 4 indicate that FGF21 promotes the phosphorylation of the SRF Ser224 site by p38 MAPK, thereby activating the SRF/MYOCD complex to increase contractile gene expression.

### FGF21 activates p38 MAPK through the KLB/FGFR1–TAK1–MKK3/6 pathway

**Fig. 5.**
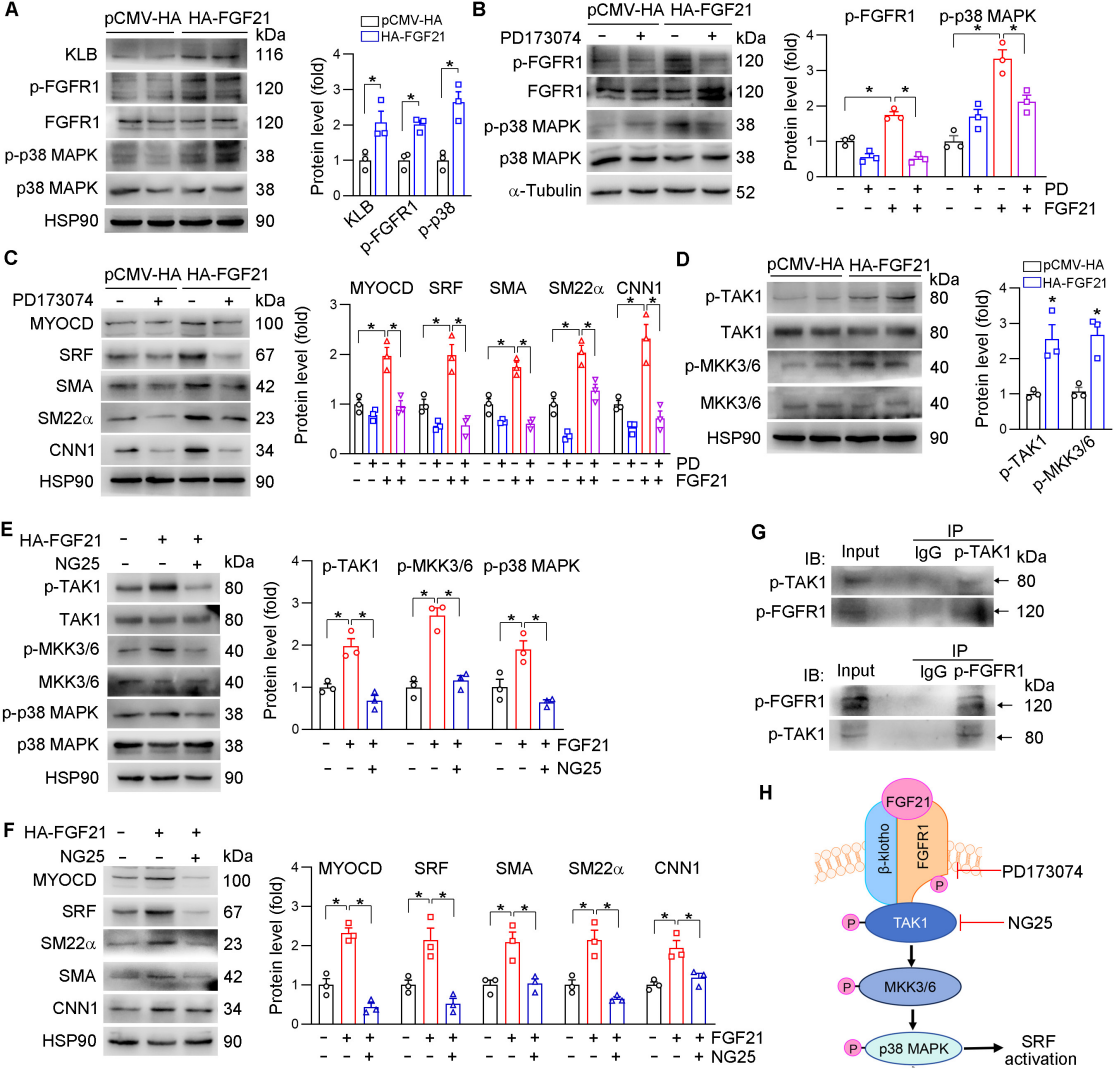
FGF21 activates p38 MAPK through the β-klotho (KLB)/FGFR1–transforming growth factor-β-activated kinase 1 (TAK1)–mitogen-activated protein kinase kinase 3/6 (MKK3/6) pathway. HASMCs were transfected with pCMV-HA or HA-FGF21 for 24 h, after which the cells were switched to complete medium for 24 h. The expression of p38 MAPK, p-p38 MAPK, FGFR1, p-FGFR1, and KLB in (A) or MKK3/6, p-MKK3/6, TAK1, and p-TAK1 in (D) was detected by Western blotting (*n* = 3). (B and C) HASMCs were transfected with pCMV-HA or HA-FGF21 for 24 h. Then, the cells were treated with PD173074 (10 nmol/l) for 24 h. The expression of p-FGFR1, FGFR1, p-p38 MAPK, and p38 MAPK (B) and MYOCD, SRF, SMA, SM22α, and CNN1 (C) was determined by Western blotting (*n* = 3). (E and F) After HA-FGF21 transfection for 24 h, HASMCs were treated with NG25 (10 μmol/l) for 24 h. The expression of p-TAK1, TAK1, p-MKK3/6, MKK3/6, p-p38 MAPK, and p38 MAPK (E) and SMA, SM22α, CNN1, MYOCD, and SRF (F) was determined by Western blotting (*n* = 3). (G) HASMCs were transfected with HA-FGF21 for 24 h, followed by culture in complete medium for 24 h. The cell lysates were subjected to immunoprecipitation with anti-p-FGFR1 or anti-p-TAK1 antibodies. The pulled-down complexes and input cell lysates were analyzed by Western blotting with the anti-p-FGFR1 or anti-p-TAK1 antibodies. (H) Diagram of FGF21-activated p38 MAPK through the KLB/FGFR1–TAK1–MKK3/6 pathway. Data information: Data are expressed as mean ± SD. Student *t* test (2 groups) or 1-way ANOVA or 2-way ANOVA followed by Tukey’s test (more than 2 groups), *P < 0.05; ns, not significant.

FGF21 exerts its biological functions through a heterodimeric receptor complex composed of FGFR1 and KLB. In our experiments, we observed that the protein level of KLB was significantly higher in HASMCs transfected with hemagglutinin (HA)-FGF21 compared to that in control HASMCs (Fig. 5A). In association with increased p-p38 MAPK by FGF21, p-FGFR1 was increased in the HA-FGF21-transfected cells (Fig. 5A). To further investigate the role of FGFR1 in FGF21 signaling, we treated the cells with PD173074, a specific inhibitor of FGFR1. We observed that PD173074 abolished the FGF21-induced increases in p-p38 MAPK and p-FGFR1 expression (Fig. 5B). Similarly, the inhibition of p-FGFR1 by PD173074 also eliminated the FGF21-induced expression of SRF, MYOCD, SMA, SM22α, and CNN1 (Fig. 5C).

The transforming growth factor-β-activated kinase 1 (TAK1)–mitogen-activated protein kinase kinase 3/6 (MKK3/6) pathway is the main upstream activator of p38 MAPK. The phosphorylation of TAK1 (p-TAK1) and p-MKK3/6 expression in HASMCs were increased by HA-FGF21 (Fig. 5D). To further determine the role of TAK1 in FGF21-induced p38 MAPK phosphorylation, we used a TAK1 inhibitor (NG25) to inhibit TAK1 phosphorylation. In association with p-TAK1 inhibition, NG25 abolished FGF21-induced p-TAK1, p-MKK3/6, and p-p38 MAPK expression (Fig. 5E). In addition, the inhibition of p-TAK1 expression abolished the effects of FGF21 on SMA, SM22α, CNN1, SRF, and MYOCD expression (Fig. 5F), indicating that the effects of FGF21 on p38 MAPK depend on TAK1 phosphorylation. To determine the mechanism of FGFR1 cross talk with the TAK1 pathway, we speculated that FGFR1 may directly interact with TAK1. Co-immunoprecipitation with antibodies against p-FGFR1 or p-TAK1 followed by immunoblotting demonstrated that these 2 proteins effectively coimmunoprecipitated (Fig. 5G). Taken together, these data demonstrate that FGF21 activates p38 MAPK through the KLB/FGFR1–TAK1–MKK3/6 pathway (Fig. 5H).

### Activation of the p38 MAPK or FGF21 analog efruxifermin attenuates intimal hyperplasia

**Fig. 6.**
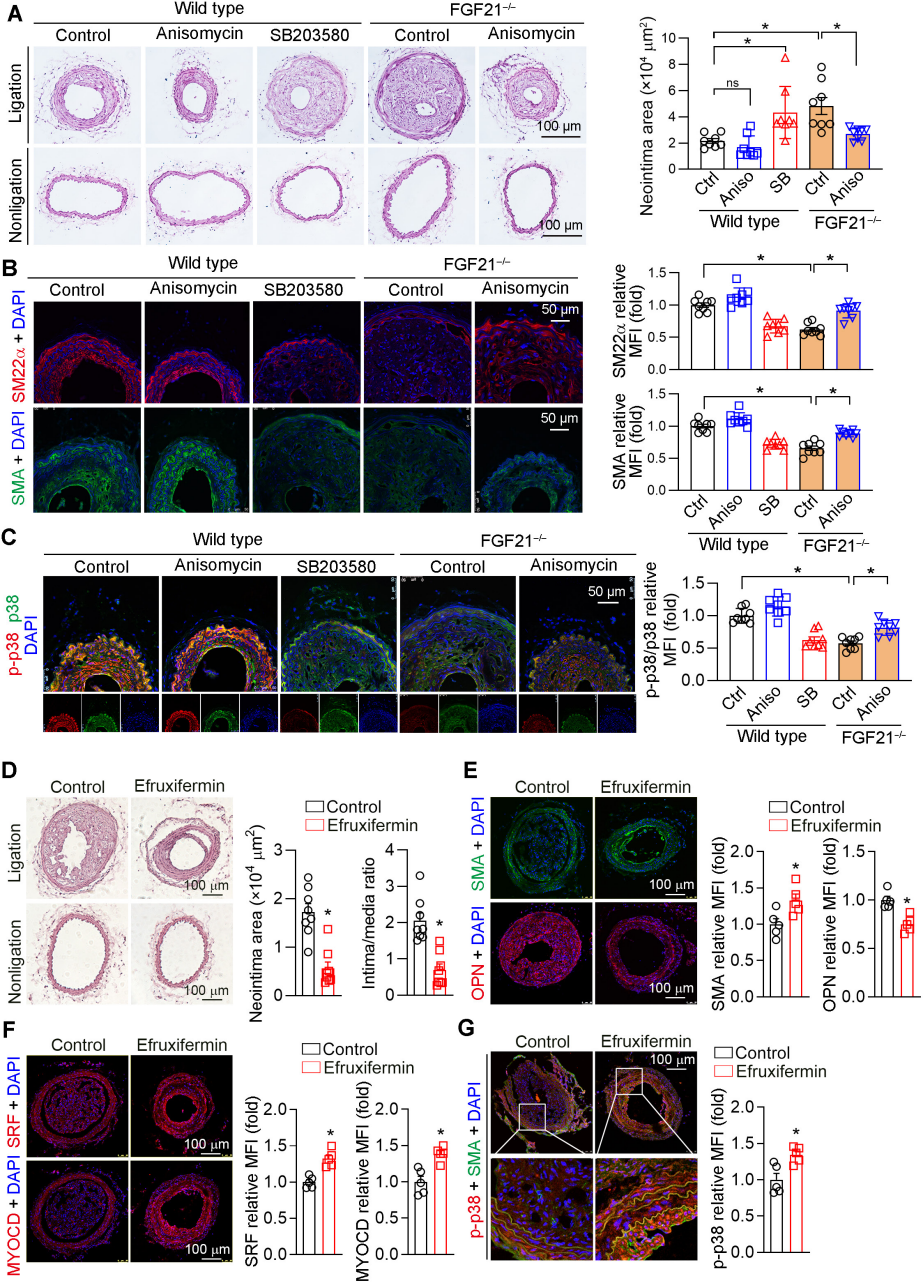
p38 MAPK activation and efruxifermin improves intimal hyperplasia. (A to C) Female C57BL/6J or FGF21^−/−^ mice (*n* = 8) were subjected to left carotid artery ligation and then intraperitoneally injected with the vehicle (distilled water), SB203580 (10 mg/kg), or anisomycin (15 mg/kg) every day, respectively, for 4 weeks. At the end of the experiment, carotid artery samples were collected and used for the following assays. HE staining for morphological analysis with quantitative analysis of neointima and media areas (A). The expression of SMA, SM22α (B), p38 MAPK, and p-p38 MAPK (C) in neointima areas was determined by immunofluorescence staining. (D to G) Left carotid artery ligation was performed on female C57BL/6J mice (*n* = 8), which were then injected subcutaneously with physiological saline or efruxifermin (5 mg/kg) once a week for 4 weeks. At the end of the experiment, carotid artery samples were individually collected and used for the following assay: (D) HE staining for morphological analysis with quantitative analysis of neointima and media areas. (E to G) The expression of SMA and OPN (E), SRF and MYOCD (F), and p-p38 MAPK in SMA-positive cells (G) in neointimal areas was determined by immunofluorescence staining (*n* = 5). Data information: Data are expressed as mean ± SD. Student *t* test (2 groups) or 2-way ANOVA followed by Tukey’s test, *P < 0.05.

The above data demonstrate that FGF21 facilitated VSMC contractile phenotypic switching by promoting the phosphorylation of p38 MAPK. To further investigate whether p38 MAPK activity has beneficial effects on neointima formation, carotid artery ligation was performed in WT or FGF21^−/−^ mice with the intraperitoneal injection of a p38 MAPK agonist (anisomycin) or a p38 MAPK inhibitor (SB203580). As shown in Fig. 6A, anisomycin reduced intimal hyperplasia in WT mice. In contrast, the p38 MAPK inhibitor SB203580 exacerbated intimal hyperplasia, which was comparable to the effect of FGF21 knockout. Anisomycin blocked FGF21 knockout-exacerbated intimal hyperplasia. In addition, immunofluorescence revealed that the expression of SMA, SM22α, SRF, MYOCD, and p-p38 MAPK was inhibited by SB203580 or FGF21 knockout, which was increased by the p38 MAPK agonist anisomycin in WT and FGF21^−/−^ mice (Fig. 6B and C and Fig. S8). These results suggest that FGF21 inhibits intimal hyperplasia by activating p38 MAPK.

Efruxifermin, an engineered Fc-FGF21 fusion protein, has been demonstrated to have beneficial effects on metabolic dysfunction-associated steatohepatitis. We demonstrated that efruxifermin reduced ligation-induced neointimal formation in a mouse model (Fig. 6D). Additionally, efruxifermin treatment reduced OPN expression and activated SMA, SRF, MYOCD, and p-p38 MAPK expression (Fig. 6E to G). Moreover, efruxifermin was administered 2 weeks post-carotid artery ligation in mice, which allowed us to evaluate its therapeutic efficacy. The HE staining results of the carotid artery demonstrated that efruxifermin attenuated intimal hyperplasia (Fig. S9). The above results suggest the therapeutic potential of efruxifermin in vascular diseases involving aberrant VSMC phenotypic changes.

## Discussion

VSMCs are highly specialized cells that play a crucial role in vasodilation and vasoconstriction, thereby regulating the inner diameter of blood vessels, blood pressure, and blood flow [9,39]. However, during the onset and progression of occlusive cardiovascular disease, VSMCs undergo phenotypic transformation into a migratory and proliferative state [40,41]. This transformation is a key event in the progression of cardiovascular diseases, including coronary heart disease [5,42,43]. Recent studies have shown that FGF21 exerts protective effects against atherosclerosis via multiple mechanisms. It can reduce liver cholesterol synthesis, increase cholesterol efflux, and limit inflammation and oxidative stress [44–47]. FGF21 has also been demonstrated to attenuate Ang II-induced vascular remodeling by regulating ACE2 in the liver [27]. FGF21 significantly inhibited intimal hyperplasia in the wire-injured common carotid artery of diabetic mice, which was associated with a reduction in high-glucose-induced NLRP3 activation [30]. FGF21 has also been shown to inhibit the osteogenic differentiation of VSMCs [48]. Despite these findings, the direct effects of FGF21 in VSMC phenotypic switching and the underlying mechanisms remain incompletely understood. Further research is needed to elucidate how FGF21 modulates VSMC behavior and to explore its potential as a therapeutic target for cardiovascular diseases.

We initially observed that FGF21 levels were substantially reduced in the neointima area of the human carotid artery. Notably, plasma FGF21 levels were lower in patients with ASCAD compared to those in non-ASCAD controls. Moreover, FGF21 was reduced in patients who developed restenosis following PCI compared to those who did not experience restenosis. Consistent with these clinical observations, we found that FGF21 expression in the aorta and serum was reduced in a mouse model of neointima formation. These data suggest that FGF21 may be involved in neointima formation. Indeed, our experiments demonstrated that FGF21 deficiency accelerated intimal hyperplasia induced by carotid artery ligation and femoral artery guidewire injury. Importantly, these effects could be rescued by FGF21 supplementation, highlighting the therapeutic potential of FGF21 in this context. In VSMCs, FGF21 promoted the transition of HASMCs from a proliferative phenotype to a contractile phenotype. This transition was characterized by increased expression of SRF, MYOCD, and contractile proteins. Additionally, FGF21 overexpression also reduced PDGF-BB-induced HASMC proliferation and migration, whereas siFGF21 increased proliferative gene expression. These data suggest that FGF21 promotes VSMC differentiation via activation of the SRF–MYOCD complex.

The human FGFR family comprises 4 distinct isoforms (FGFR1 to FGFR4) and belongs to the receptor tyrosine kinase superfamily [49,50]. FGFR1 interacts with FGF ligands, initiating the dimerization of extracellular receptor domains, transphosphorylation of the intracellular kinase domain, and subsequent activation of downstream signaling pathways, such as the MAPK and phosphoinositide-3-kinase/AKT pathways [51,52]. FGF/FGFR signaling plays a pivotal role in diverse physiological processes, including cell proliferation, migration, and survival [51]. FGFR1 has been demonstrated to affect the VSMC phenotype. FGF2 synergizes with PDGF-BB to promote VSMC proliferation, which requires the formation of the PDGFRβ–FGFR1–FRS2 complex [53]. Surprisingly, we found that FGF21 can bind and activate FGFR1, leading to the inhibition of VSMC proliferation and increasing contractile gene expression. The distinct outcomes of FGFR1 activation may depend on the different subsequent effectors. Previous studies have established that the activation of the NF-κB pathway by FGFR1 is dependent on the participation of TAK1 [54]. This finding implies a potential molecular mechanism underlying the plausible interaction between FGFR1 and TAK1. Our experimental data clearly demonstrated that FGFR1 can directly interact with TAK1, thereby governing the phenotypic transformation of VSMCs. Furthermore, inhibition of FGFR1 or TAK1 via specific antagonists effectively blocked FGF21-induced SRF, MYOCD, and contractile gene expression.

TAK1 is known to activate the MKK3/6 pathway to induce p38 MAPK phosphorylation [55–58]. Previous reports have also shown that MKK3/6 may regulate the migration of SMCs by controlling p38 MAPK [59,60]. These findings suggest that MKK3/6 plays a significant role in the molecular mechanism underlying the phenotypic transformation of VSMCs. However, it appears contradictory that inhibiting MKK3/6 and downstream p38 MAPK leads to the inhibition of VSMC migration. We determined that FGF21 can increase the activation of p-MKK3/6 and p-p38 MAPK, thereby inhibiting VSMC migration and increasing the contractile phenotype transition. Recent studies have reported that p38 MAPK regulates the phenotypic transition of VSMCs by modulating the expression of SRF and MYOCD [61,62]. FGF12 promoted the expression of SRF and MYOCD by activating the p38 MAPK pathway, thereby inhibiting the phenotype conversion and cell cycle progression of HASMCs [20]. Similarly, 15(s)-hydroxytetradecanoic acid inhibited the proliferation and migration of VSMCs by promoting the phosphorylation of ERK1/2, JNK, and p38 MAPK, thereby alleviating balloon injury-induced intimal hyperplasia [63]. Therefore, p38 MAPK is closely related to the phenotypic transformation of VSMCs. p38 MAPK is reported to induce MYOCD expression by activating myocyte enhancer factor 2, an upstream transcription factor of MYOCD [61]. However, the detailed mechanism of p-p38 MAPK-induced SRF/MYOCD activation is not fully understood. We predicted that SRF, but not MYOCD, was a direct substrate of p-p38 MAPK. Indeed, we determined that p-p38 MAPK phosphorylated SRF at serine 224, thereby enhancing the transactivation of the SRF/MYOCD complex. While SRF may be phosphorylated by GSK3β at serine 224 in endothelial cells or neurons [64,65], our study found that neither p-GSK3β nor GSK3β was affected by FGF21. Therefore, SRF may be phosphorylated by different enzymes at serine 224, which results in distinct outcomes in VSMC phenotypic transition.

This study provides important insights into the potential of FGF21 as a target for the treatment of diseases associated with the phenotypic switching of SMCs. However, some limitations should be noted. First, we used FGF21^−/−^, FGF21^HepKO^, and KLB^SMKO^ mice to determine the effect of FGF21 on neointima formation; FGF21 VSMC-specific knockout mice would provide more precise insights into the underlying mechanisms. Second, the SM22-Cre driver can also confer expression in nonmuscular cells including perivascular adipocytes and their precursors, myeloid cells, and platelets. In subsequent experiments, we plan to obtain KLB^flox/flox^Myh11-Cre and FGF21^flox/flox^Myh11-Cre mice to verify the direct effect of FGF21 on the contraction phenotype of VSMCs. Third, we used female mice only in this study. Although we used HASMCs from both sex in vitro experiments, we cannot rule out the possibility that male mice may response differently to FGF21 compared with female mice.

In conclusion, we revealed the functional role of FGF21 in neointima formation after carotid artery injury. FGF21 promotes the differentiation of VSMCs through p38 MAPK-promoted SRF phosphorylation, thereby activating the SRF/MYOCD complex. Moreover, our results indicate that an FGF21 analog (efruxifermin) or a p38 MAPK agonist prevents the formation of intimal hyperplasia induced by carotid artery ligation, suggesting that manipulating FGF21 expression can exert therapeutic effects in vascular diseases involving aberrant VSMC phenotypic changes.

## Materials and Methods

The detailed methods are available in the Supplementary Materials.

### Ethical compliance

The study with human samples was approved by the Ethical Review Board of the First Affiliated Hospital of University of Science and Technology of China (2019KY165) or the Ethical Committee of Tianjin Baodi Hospital (202002) and adhered strictly to the Declaration of Helsinki Principle 2008. All samples were collected after written informed consent was obtained from patients or their family members.

The protocols for in vivo studies with mice were approved by the Ethics Committee of Hefei University of Technology (HFUT20200201001) and conformed to the Guide for the Care and Use of Laboratory Animals published by the National Institutes of Health. The animal studies were reported in compliance with the ARRIVE guidelines.

The mice were housed in specific-pathogen-free units of the Animal Center at Hefei University of Technology (with a 12-h light cycle from 8 AM to 8 PM, 23 ± 1 °C, 60% to 70% humidity) and maintained on a standard rodent diet with free access to water in plastic bottles. The mice were allowed to acclimatize to their housing environment for at least 7 d before the experiments. For carotid artery ligation or femoral artery guidewire injury, anesthesia was induced using 100% O_2_/4% isoflurane and maintained throughout the surgery by the administration of 100% O_2_/2% isoflurane. Buprenorphine (0.1 mg/kg) was subcutaneously injected for preemptive analgesia. At the end of the experiment, the mice were euthanized by intraperitoneal injection of an overdose of pentobarbital (500 mg/kg).

### Analysis of FGF21 or SM22α in human samples

We collected samples from 3 patients with carotid artery stenosis whose atherosclerotic intima was surgically removed at the First Affiliated Hospital of University of Science and Technology of China. Symptomatic patients had proven indications (one or more transient ischemic attacks in the past 6 months and carotid stenosis ≥70% or mild stroke within 6 months and carotid stenosis ≥70%) for carotid endarterectomy according to the guidelines from the American Heart Association [66].

To determine whether FGF21 expression affects ISR disease, we collected blood samples from 22 patients who had undergone PCI at the First Affiliated Hospital of University of Science and Technology of China, among whom 11 patients experienced significant ISR (defined as >50% stenosis within the stent or 5 mm from the edge of the stent using coronary angiography) after 1 year of follow-up.

To determine the plasma FGF21 level in ASCAD patients, we collected 28 blood samples from those who were diagnosed with ASCAD according to the 2018 JCS Guide “Diagnosis and Treatment of Acute Coronary Syndrome” at Tianjin Baodi Hospital. The participants in the non-ASCAD group (*n* = 28) were collected from those who were hospitalized with symptoms such as chest stuffiness or thoracalgia, but no severe coronary stenosis was observed by coronary angiography. The inclusion criteria were as follows: (a) stenosis of the left main stem, left anterior descending branch, left circumflex branch, and right coronary artery and its large branches; if the patients’ stenosis was ≥50%, then they were diagnosed with coronary heart disease and were included in the coronary artery atherosclerosis group (ASCAD); (b) symptoms such as chest stuffiness or thoracalgia but no severe coronary stenosis observed by coronary angiography; these patients were included in the non-ASCAD group; (c) age ≥40 years; (d) ability to express their inner thoughts fluently; and (e) available complete patient data for clinical research. Sex and age were matched between the 2 groups via random sampling. The exclusion criteria were as follows: (a) previous PCI; (b) severe congestive heart failure, severe liver insufficiency, infection, or other diseases (malignant tumors and organ grafting); (c) left ventricular ejection fraction less than 35%; and (d) history of drug abuse.

Carotid artery samples were fixed, dehydrated, and embedded in paraffin, and 5-μm paraffin sections were prepared for the determination of FGF21 and SM22α expression by immunofluorescence staining. We isolated plasma from blood samples, and the FGF21 level was measured using an ELISA kit (Cat. No. E-EL-H0074, Elabscience Biotechnology Co., Ltd.).

### Animals and the neointima formation model

C57BL/6J WT mice and FGF21-deficient (FGF21^−/−^) mice with the same background as the WT mice were purchased from GemPharmatech (Nanjing, China). FGF21^flox/flox^ and Alb-Cre mice were kindly provided by Dr Suowen Xu from the University of Science and Technology of China. KLB^flox/flox^ and SM22-Cre mice were purchased from Cyagen (Suzhou, China). All mice were bred at the Animal Center of the Hefei University of Technology (Hefei, China).

For analysis of the effects of FGF21 or its analog efruxifermin (Cat. No.: HY-P99930, MedChemExpress) on neointima formation, carotid artery ligation in mice was performed as previously described [67]. Briefly, female mice (~8 weeks old) were anesthetized by isoflurane inhalation (induced with 100% O_2_/4% isoflurane and maintained with 100% O_2_/2% isoflurane) and injected with buprenorphine (0.1 mg/kg) subcutaneously for preemptive analgesia. All mice were then subjected to carotid injury by left carotid artery ligation with 6-0 silk sutures. The right carotid artery underwent a sham operation without ligation. After surgery, the mice were administered the corresponding treatments and the diet was continued for another 4 weeks.

To analyze the effect of FGF21 on neointima formation, the femoral arteries of 8-week-old female FGF21^−/−^ and C57BL/6J mice were injured by endoluminal passage of an angioplasty guidewire as previously described [68]. Arteries on one side were not injured and served as negative controls. Briefly, the mice were anesthetized with inhaled isoflurane (induced with 100% O_2_/4% isoflurane and maintained with 100% O_2_/2% isoflurane) and injected with buprenorphine (0.1 mg/kg) subcutaneously for preemptive analgesia. The femoral arteries were exposed by a longitudinal groin incision and viewed under a surgical microscope. The distal portion of the artery was encircled with a 6-0 silk suture, a vascular clamp was placed proximally at the level of the inguinal ligament, and a 0.01-inch-diameter guidewire (7515873, William Cook Europe ApS, USA) was introduced into the arterial lumen through an arteriotomy made in the distal perforating branch. After the release of the clamp, the guidewire was advanced to the level of the aortic bifurcation and allowed to denude the endothelium for 3 min. After the wire was removed, the arteriotomy site was ligated, and the skin was closed. After surgery, the mice were administered the corresponding treatments and the diet was continued for another 4 weeks.

### Data analysis

All experiments were repeated at least 3 times (biological replicates), and the representative results are presented. The results are expressed as mean ± SD. The raw data were initially subjected to a normal distribution analysis with the SPSS software (one-sample Kolmogorov–Smirnov nonparametric test). All normally distributed data were then analyzed using an unpaired Student *t* test (2 groups), 1-way analysis of variance (ANOVA), or 2-way ANOVA followed by Tukey’s test (more than 2 groups) by the GraphPad Prism software (version 8.0, GraphPad Software, San Diego, CA). Differences were considered significant if *P* < 0.05. For human samples, the Pearson chi-square test was used to compare sex, smoking status, hypertension status, and diabetes status between the non-ASCAD group and the ASCAD group, and Fisher’s exact test was used to compare the same variables between the PCI without restenosis group and the PCI with restenosis group.

## Data Availability

The data that support the findings of this study are available from the corresponding authors on reasonable request.
