# Supplementary material for: Fibroblast Growth Factor 21 Promotes Vascular Smooth Muscle Cell Contractile Polarization via p38 Mitogen-Activated Protein Kinase-Promoted Serum Response Factor Phosphorylation
Source: Research (Wash D C). 2025 Aug 5;8:0815. doi: 10.34133/research.0815 (PMC12324144; doi:10.34133/research.0815)
Supplement: Supplementary 1 — Supplementary Materials and Methods Figs. S1 to S9 Tables S1 to S3 [file research.0815.f1.docx]

**Supporting Information**

**I.** **Supplementary materials and methods**

**Reagents**

FGF21 ELISA Kit (Cat#: E-EL-H0074) was purchased from Elabscience Biotechnology Co.,Ltd. (Wuhan, China). Cell Contraction Assay Kit (Cat#: CBA-5020) was purchased from Cell Biolabs (California, USA). PDGF-BB (Cat#: 10572-H07Y), rhFGF21 (Cat#:10911-HNAE) and rmFGF21 (Cat#: 50421-M08H) were purchased from Sino Biological Inc. (Beijing, China). Heparin (Cat#: HY-17567), SB203580 (Cat#: HY-10256), PD173074 (Cat#: HY-10321), anisomycin (Cat#: HY-18982) and efruxifermin (Cat#: HY-P99930) were purchased from MedChemExpress (Monmouth Junction, NJ, USA). NG25 (Cat#: S8868) was purchased from Selleck (Shanghai, China). HA tag Nanoab or control Magnetic Beads was purchased from NuoyiBio (Shanghai, China). HE Stain Solution (One Step Method) (Cat#: G4520) was purchased from Solarbio Science & Technology Co., Ltd (Beijing, China). The antibodies used were listed in Online Supplementary Table S1.

**Cell culture**

Human aortic smooth muscle cells (HASMCs) were purchased from ATCC (Rockville, MD) and cultured in complete DMEM medium containing 10% fetal calf serum and 50 μg/mL of penicillin/streptomycin. Cells less than 10 passages were used for experiments, in which the cells were fasted for 12 h before the corresponding treatments in serum-free medium. All studies with HASMC were approved by the Ethics Committee of Hefei University of Technology and adhered strictly to the Declaration of Helsinki Principle 2008.

***In vivo* studies with mice**

To study the metabolic effect of FGF21 on neointima formation, female FGF21^f/f^ and FGF21^HepKO^ mice (~8-week-old) were performed carotid artery ligation. After 4 weeks, the aorta samples were collected.

To study the effect of rmFGF21 on neointima formation, carotid artery ligation in mice was performed. female C57BL/6J and FGF21^-/-^(~8-week-old) were divided into 3 groups (8 mice/group), The experimental groups are as follows: group 1, C57BL/6J; group 2, FGF21^-/-^; group 3, FGF21^-/-^+rmFGF21. After surgery described above, FGF21^-/-^ mice were injected with murine-derived recombinant FGF21 protein (rmFGF21, 600 μg/kg) twice a week in caudal vein. After 4 weeks, the aorta samples were collected.

VSMC-specific β-klotho knockout mice (KLB^f/f^SM22-Cre, KLB^SMKO^) were conducted to disclose the direct effect of FGF21 on intimal hyperplasia. Female KLB^flox/flox^ (KLB^f/f^) and KLB^SMKO^ mice (~8-week-old) were divided into 4 groups (6 mice/group), and performed left carotid artery ligation. The experimental groups are as follows: group 1, KLB^f/f^; group 2, KLB^f/f^+rmFGF21; group 3, KLB^SMKO^; group 4, KLB^SMKO^ +rmFGF21. After surgery described above, murine-derived recombinant FGF21 protein (rmFGF21, 600 μg/kg) were injected twice a week in caudal vein. After 4 weeks, the aorta samples were collected.

To study the effect of p38 MAPK on neointima formation, carotid artery ligation in mice was performed. Female C57BL/6J and FGF21^-/-^(~8-week-old) were divided into 5 groups (8 mice/group): group 1, C57BL/6J; group 2, C57BL/6J+SB203580; group 3, C57BL/6J+Anisomycin; group 4, FGF21^-/-^; group 5, FGF21^-/-^+anisomycin. After surgery described above, mice were injected intraperitoneally once every two days with anisomycin (15 mg/kg) or SB203580 (10 mg/kg). After 4 weeks, the aorta samples were collected.

To study the effect of efruxifermin on neointima formation, female C57BL/6J mice (n=8) were performed left carotid artery ligation, and then injected subcutaneously with physiological saline or efruxifermin (5 mg/kg) once a week for 4 w. At the end of experiment, carotid artery samples were individually collected. In the therapeutic intervention model, animals received subcutaneous injections of efruxifermin (5 mg/kg) or vehicle control 2 weeks post-carotid artery ligation, with carotid artery samples harvested at week 4 for analysis.

At the end of the experiment, the mice were euthanized by i.p injection of an overdose of pentobarbital (500 mg/kg). Then, mouse left and right carotid arteries were collected and used to prepare 5-μm frozen sections. The sections were then used to detect neointima areas by hematoxylin & eosin (HE) staining. The images were captured and used to determine the neointima and media areas (μm^2^/section) using the Photoshop CS3 software.

**Immunofluorescence staining**

Expression of FGF21, SMA, SM22α, OPN, p38 MAPK, p-p38 MAPK, SRF and MYOCD in carotid artery or femoral artery were determined by immunofluorescence staining of the 5-μm frozen sections of the corresponding samples and primary antibodies as described^1^. We have obtained all the fluorescence images in the same panel with same parameters. Then, the images were analyzed with ImageJ to obtain mean fluorescence intensity (MFI). In order to clearly see the changes of the corresponding treatments, all MFI were normalized with the MFI in the control group. The relative MFI was used as Y-axis for quantitative analysis the immunofluorescence images^2^.

**Determination of cell viability and cell migration**

The 3-(4,5-dimethylthiazol-2-yl)-2,5-diphenyltetrazolium bromide (MTT) colorimetric assay was used to determine cell viability of HASMC. Briefly, cells in 96-well plate were treated with HA-FGF21 or siFGF21 in serum-free medium. After treatment and aspiration of the treatment medium, cells in each well were added with 100 μL MTT solution (5 mg/mL, dissolved in serum-free medium) and incubated for 4 h. After careful removal of liquid, each well was added with 150 μL DMSO and incubated for 10 min with gentle shake on an orbital shaker to dissolve the purple formazan crystals formed within cells completely. The absorbance of solution at 570 nm in each well was determined by a microplate reader (BioTek, USA). The results were normalized to the mean value of control group and expressed as % of control.

HASMC were wounded using a plastic tip and washed twice with PBS to remove the suspended cells or cell debris. After adding with medium containing 2% FBS, cells were photographed under a light microscope (Leica, Wetzlar, Germany) and the width of scratching was recorded as W_0_. Cells were then received indicated transfection, and continued in culture for 24 h followed by photograph and recording the width of scratching uncovered as W_24_. The migration rate was calculated as (W_0_ – W_24_)/W_0_ × 100%.

**Preparation of FGF21 expression vector or siRNA transfection**

The cDNA encoding human FGF21 was generated by reverse transcription (RT) followed by PCR using total RNA extracted from HASMC and the following primers: forward, 5′-AAATCTAGAGATGGACTCGGACGAGACCGGTGG-3′; backward: 5′-CCACTCGAGACAGGAAGCGTAGCTGGGGCCTGC-3′. The RT-PCR product was digested with XbaI and XhoI, and then subcloned into an expression vector pCMV-HA. The plasmid was named as HA-FGF21 after the sequence and protein expression were confirmed. Cells at ~60% confluence in 6-well plates were transfected with pCMV-HA empty vector or HA-FGF21 using Lipofectamine® RNAiMAX Transfection Reagent (Invitrogen) for 24 h, cells were switched into complete DMEM medium and cultured for another 24 h or then received the indicated treatment.

The FGF21 siRNA and control siRNA were purchased from RiboBio (Guangzhou, China). HASMC in 6-well plates were transfected with si-control or FGF21 siRNA using Lipofectamine® RNAiMAX Transfection Reagent (Invitrogen). After 24 h of transfection, cells were switched into complete DMEM medium and cultured for another 24 h or then received the indicated treatment.

**Western blotting and quantitative real-time PCR (qPCR)**

After the indicated treatment, Protein expression in total proteins from tissue samples or cells were determined by Western blotting. Briefly, total protein was extracted from HASMC or tissue samples with the RIPA lysis buffer. The protein samples were then separated by SDS-PAGE and transferred onto NC membrane. The membranes were blocked with 5% skim milk in PBST for 1 h at room temperature. Then, according to the molecule weight of the target protein, the membrane was cut into smaller pieces prior to hybridization. The membrane was then separately incubated with specific primary antibody overnight at 4°C. After washed with PBST for three times, the membrane was incubated with HRP-conjugated secondary antibodies for 1 h at room temperature. Membrane was captured with chemiluminescence imaging system (Qinxiang, ChemiScope 3300 Mini, China). The densitometric values of immunoreactive bands were measured using Image J. The density of target band was normalized to HSP90 in the corresponding sample to reduce variance.

After treatment, total RNA was extracted from cells using Trizol reagent (Invitrogen, Carlsbad, CA, USA). Levels of MYH11, CNN1, SMA, SM22α, SRF and MYOCD in cells were analyzed by qPCR with primers listed in the Online Supplementary Table S3. For qPCR, the target gene level was normalized by GAPDH mRNA in the corresponding samples.

**Co-immunoprecipitation (co-IP) assay**

The interaction of SRF, MYOCD and p-p38 MAPK was determined by co-IP. Briefly, HASMC were transfected with HA-MYOCD for 24 h in serum-free medium, followed by switching the cells into complete medium to culture for another 24 h. After protein extraction, anti-SRF, p-p38 MAPK, MYOCD or IgG antibody was incubated with a protein A/G magnetic beads overnight, followed by washing with PBST. Then, the protein extraction was added and incubated for 1 h, followed by washing with PBST. The proteins binding with beads were eluted with 2 × SDS loading buffer. The corresponding proteins were measured by Western blot.

293Ts were transfected with HA-SRF or pCMV-HA for 24 h in serum-free medium, followed by switching the cells into complete medium to culture for another 24 h. After protein extraction, the cell lysate was added to HA tag -Nanoab-Magnetic Beads (30 μL) for 3 h. After washed with PBS for 3 times, the proteins were pulled-down with 2 × SDS loading buffer, and the eluent and input cell lysates were used to analyze HA (represent SRF), p-Serine or p-Threonine expression by Western blotting.

**ChIP-qPCR assay**

HASMCs were treated with or without rhFGF21 (0.5 mg/mL) for 24 h. Then, the ChIP-qPCR was performed as described previously^3^. Briefly, the cells were cross-linked by addition of formaldehyde followed by sonication in a lysis buffer [50 mM Hepes-KOH (pH 7.5), 140 mM NaCl, 1% Triton X-100, 1 mM EDTA, 0.1% sodium deoxycholate, 0.1% SDS and the protease inhibitors of aprotinin/leupeptin (0.08 mM and 0.1 mM respectively)] to fragment DNA into an average size of 500-1000 bp. The input PCR was conducted with DNA extracted from the sonicated chromatin after reversal of the cross-linking. Based on the results of input, we conducted immunoprecipitation with the same amount of chromatin from each sample and anti-SRF or normal IgG, separately. The binding of MYOCD-SRF complex with the SRE in SMA or SM22α promoter region was determined by qPCR. The primers for the ChIP assays were: SMA SRE forward, 5’-CCACAGGCGGCTGAACCGCC-3’, and reverse, 5’-GGCTGGGCTGCTCCACACT-3’; SM22α SRE forward, 5’-CCCGCTCCATCTCCAAAGCAT-3’, and reverse, 5’-CACTGGCTCGCTCCTCG-3’, respectively.

**Determination of SRF phosphorylation by LC-MS/MS**

HA-SRF was overexpressed in HASMCs for 24 h in serum-free medium, followed by switching the cells into complete medium with anisomycin (10 μmol/L) to culture for 24 h. After SDS-PAGE electrophoresis, immerse the gel in a dye solution containing Coomassie brilliant blue G-250 and incubate it for 2 hours with gentle shaking at room temperature. Subsequently, remove the dye solution and immerse the gel in a decolorizing solution, gently shaking it for 2 hours. Discard the decolorizing solution and replace it with fresh decolorizing solution, continuing the decolorization process until clear blue bands and a clean background are achieved. Once the bands of interest are clearly visible, carefully excise the band from the gel.

For in-gel tryptic digestion, gel pieces were destained in 50 mM NH_4_HCO_3_ in 50% acetonitrile (v/v) until clear. Gel pieces were dehydrated with 100 μL of 100% acetonitrile for 5 min, the liquid removed, and the gel pieces rehydrated in 10 mM dithiothreitol and incubated at 56 °C for 60 min. Gel pieces were again dehydrated in 100% acetonitrile, liquid was removed and gel pieces were rehydrated with 55 mM iodoacetamide. Samples were incubated at room temperature, in the dark for 45 min. Gel pieces were washed with 50 mM NH_4_HCO_3_ and dehydrated with 100% acetonitrile. Gel pieces were rehydrated with 10 ng/μL trypsin resuspended in 50 mM NH_4_HCO_3_ on ice for 1 h. Excess liquid was removed and gel pieces were digested with trypsin at 37 °C overnight. Peptides were extracted with 50% acetonitrile/5% formic acid, followed by 100% acetonitrile. Peptides were dried to completion and resuspended in 2% acetonitrile/0.1% formic acid.

The tryptic peptides were dissolved in 0.1% formic acid (solvent A), directly loaded onto a home-made reversed-phase analytical column on an EASY-nLC 1000 UPLC system. The peptides were subjected to NSI source followed by tandem mass spectrometry (MS/MS) in Q ExactiveTM Plus (Thermo) coupled online to the UPLC. The electrospray voltage applied was 2.0 kV. The m/z scan range was 350 to 1800 for full scan, and intact peptides were detected in the Orbitrap at a resolution of 70,000.

**II. Supplementary figures and figure legends**


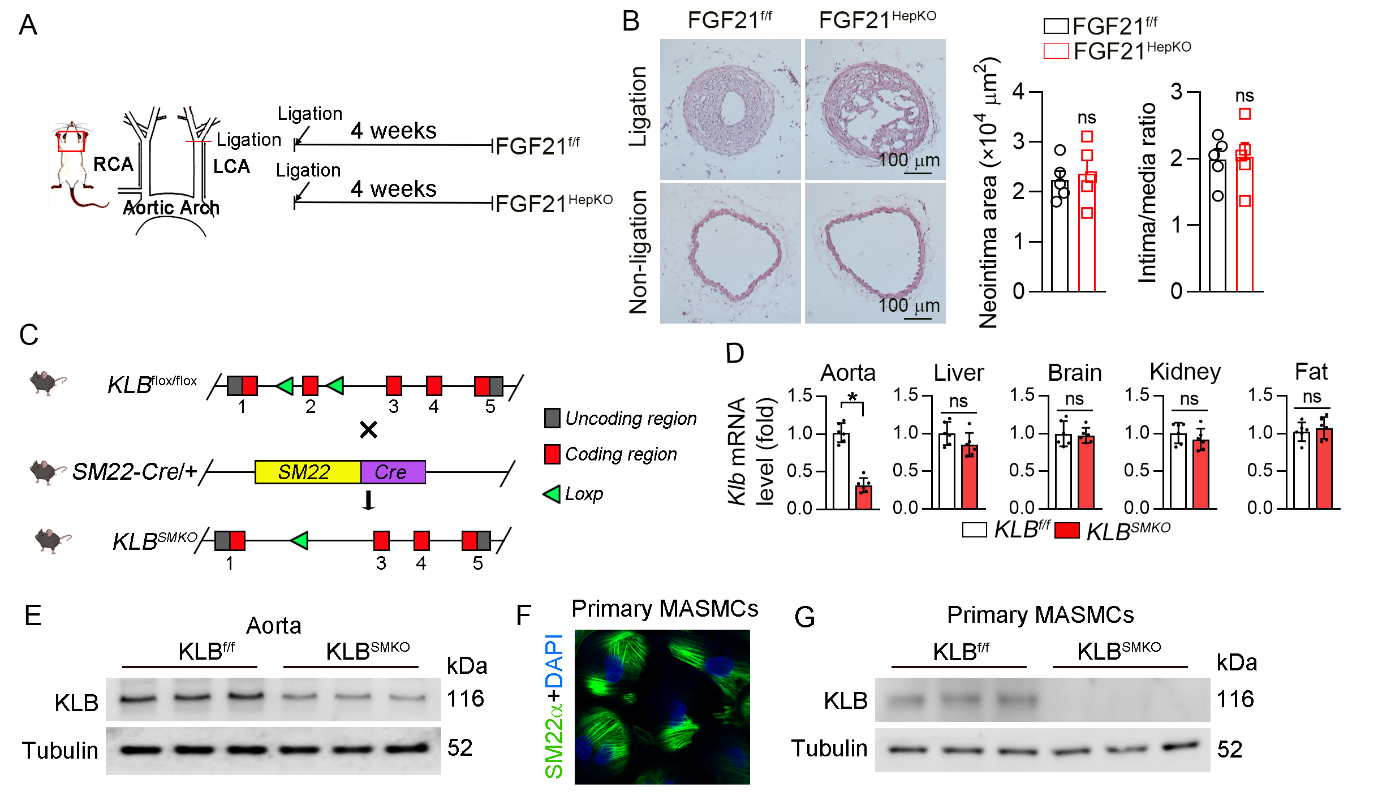


**Figure S1. Hepatocyte-specific knockout of FGF21 does not affect intimal hyperplasia**

(**A, B**) Carotid artery ligation was performed in female FGF21^f/f^ and FGF21^HepKO^ mice. Both the left and right carotid arteries were collected, and cross sections were prepared for HE staining for morphological analysis with quantitative analysis of the neointima and media areas (n=5). (**C**) Construction of VSMC-specific β-klotho knockout mice. (**D**) KLB mRNA level in the aorta, liver, brain, kidney and fat was determined by qPCR (n=6). (**E**) Aortas were harvested from KLB^f/f^ and KLB^SMKO^ mice, protein was extracted and KLB expression was detected by Western blotting (n=3). (**F, G**) Primary MASMCs were isolated from KLB^f/f^ and KLB^SMKO^ mice, immunofluorescence staining for SM22α expression was performed (**F**); KLB expression in primary MASMCs was detected by Western blotting (**G**) (n=3). Data information: Data are expressed as the means ± SDs. Student’s t-test, **P*<0.05. ns: not significant difference.


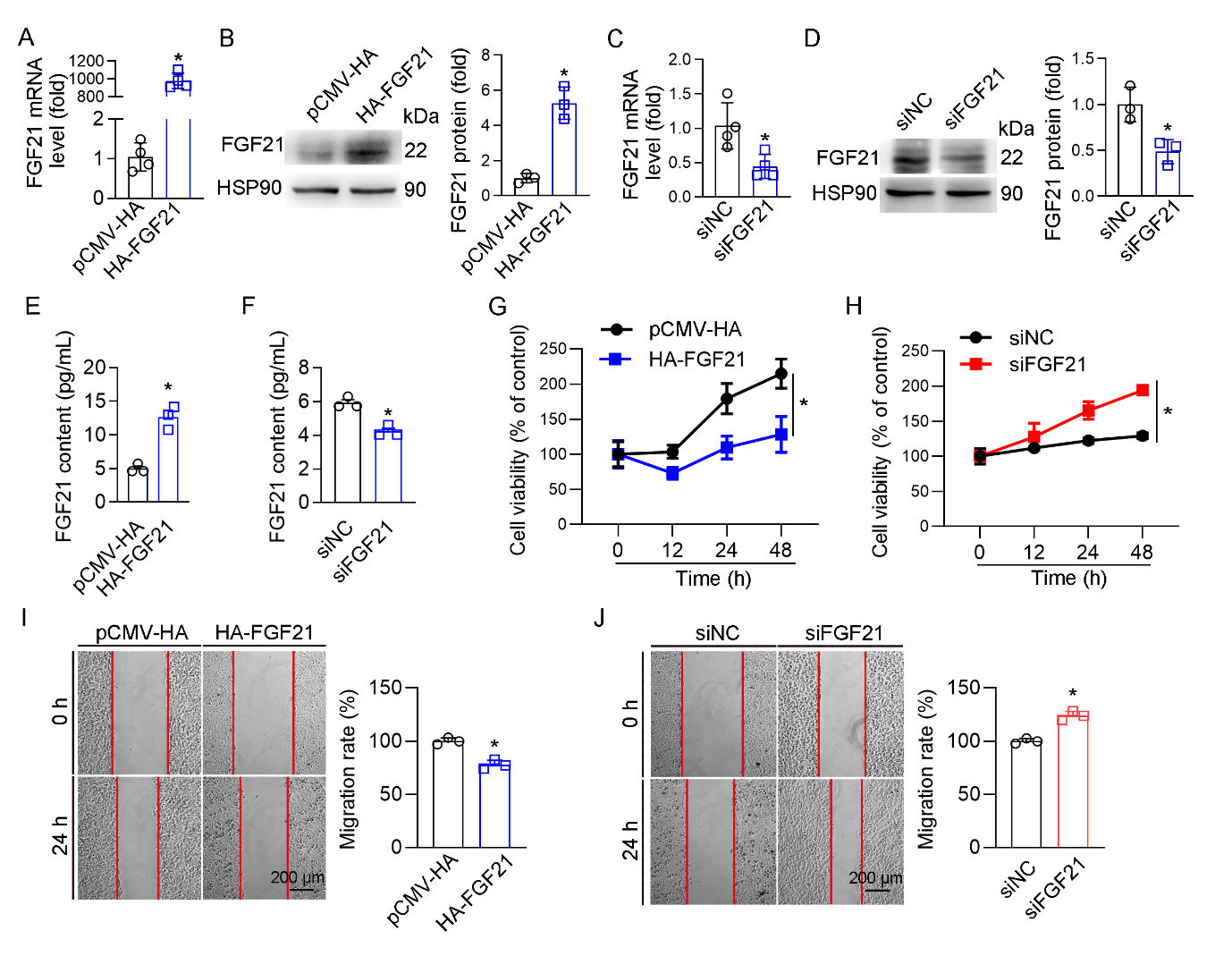


**Figure S2. FGF21 inhibits the proliferation and migration of HASMCs**

(**A-F**) HASMCs were transfected with pCMV-HA/HA-FGF21 or siNC/siFGF21 for 12 h. After culture for another 24 h, the expression of FGF21 was determined by Western blotting (**B, D**, n=3) or qPCR (**A, C**, n=4). FGF21 content in culture medium supernatants was detected by ELISA assay (**E, F**). (**G, H**) HASMCs were transfected with siNC/siFGF21 or pCMV-HA/HA-FGF21 for 12 h, then cultured in fresh medium, and the viability of the cells was determined via MTT assays (n=8). (**I, J**) HASMCs in 12-well plates were transfected with pCMV-HA/HA-FGF21 or siNC/siFGF21 for 12 h, then cultured in fresh medium supplemented with 2% FBS, and a migration assay was conducted via a cell scratch test. The photos were taken at the beginning and end of the scratch test. The width of the scratch at 0 h or 24 h was recorded as W_0_ or W_24_. The migration rate was calculated as (W_0_ – W_24_)/W_0_ × 100%. Data information: Data are expressed as means ± SDs. Student’s t-test, **P*<0.05. Data information: Data are expressed as the means ± SDs. Student’s t-test, **P*<0.05.


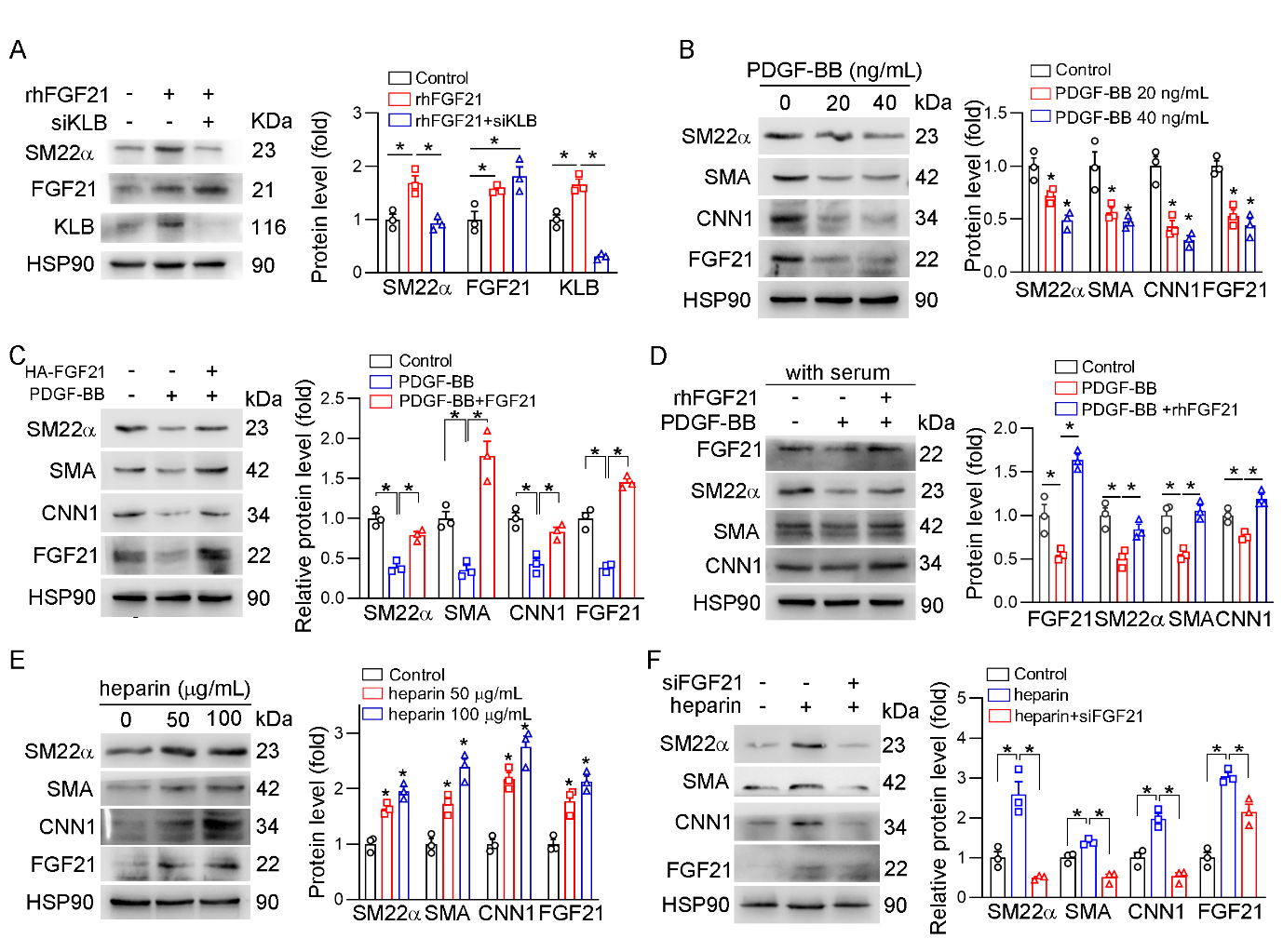


**Figure S3. FGF21 increases contractile gene expression**

(**A**) HASMCs were transfected with siNC/siKLB for 24 h, followed by treatment with or without rhFGF21 (0.5 mg/mL) for 18 h, and SM22α, FGF21 and KLB expression was detected by Western blotting (n=3). (**B, E**) HASMCs were treated with PDGF-BB (**B**) or heparin (**E**) at the indicated concentrations for 24 h. The protein expression of SM22α, SMA, CNN1, and FGF21 was determined by Western blotting (n=3). (**C, F**) HASMCs were transfected with pCMV-HA/HA-FGF21 or siNC/siFGF21 for 12 h. Then, the cells were treated with PDGF-BB (40 ng/mL) or heparin (100 μg/mL) for 24 h. FGF21, SMA, CNN1 and SM22α protein expression was determined by Western blotting (n=3). (**D**) HASMCs were treated with PDGF-BB (40 ng/mL) or plus rhFGF21 (0.5 mg/mL) for 18 h in complete medium. The expression of FGF21, SM22α, SMA and CNN1 was determined by Western blotting (n=3). Data information: Data are expressed as the means ± SDs. One-way ANOVA followed by Tukey’s test, **P*<0.05.


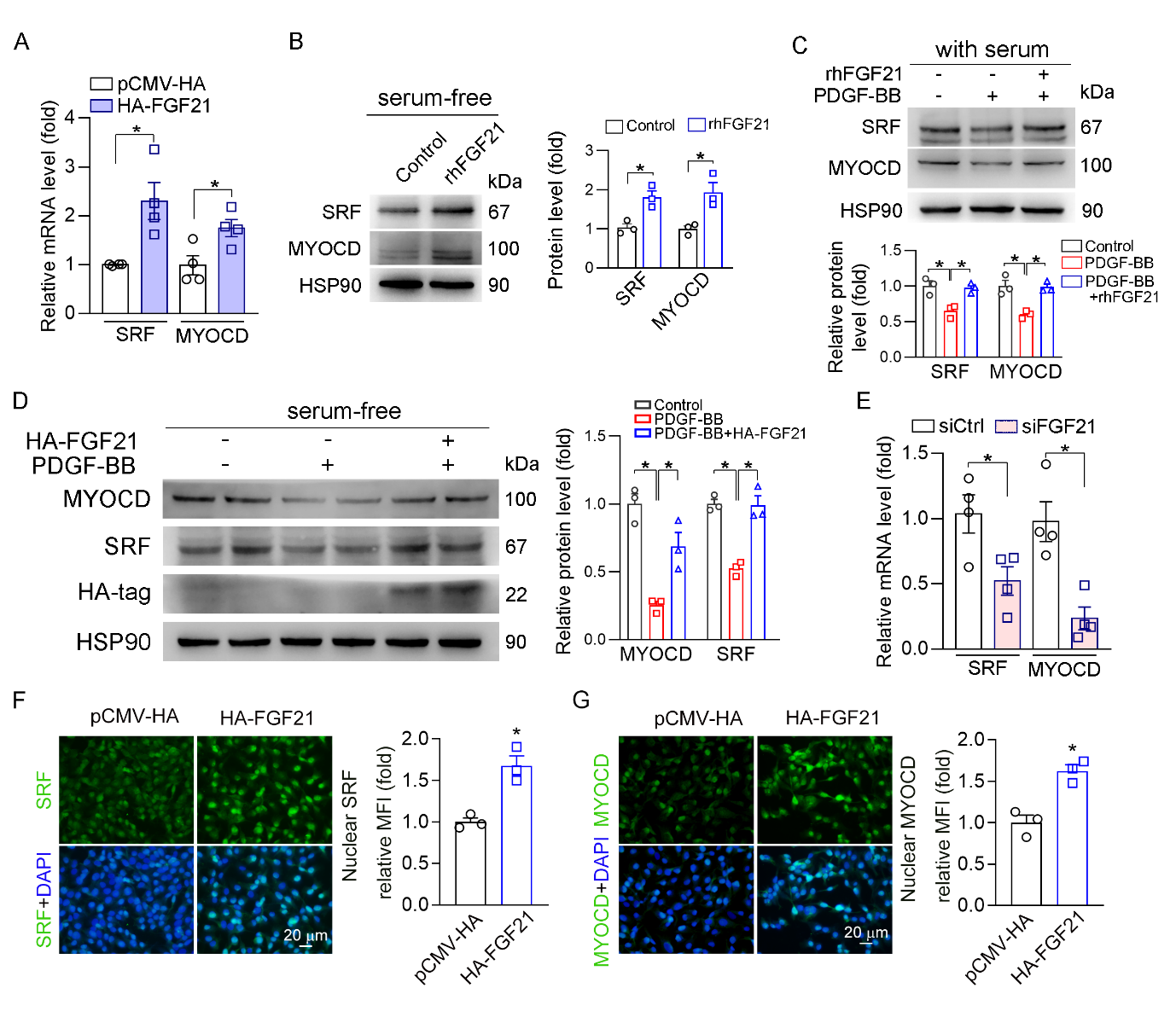


**Figure S4.** **FGF21 increases SRF and MYOCD expression in HASMCs**

(**A, E**) HASMCs were transfected with HA-FGF21 (**A**) or FGF21 siRNA (**E**) for 12 h. After cultured in complete medium for another 24 h, total RNA was isolated and subjected to determine SRF and MYOCD mRNA expression by qPCR (n=4). (**B**) HASMCs were treated with rhFGF21 (0.5 mg/mL) for 18 h. The protein expression of SRF and MYOCD was determined by Western blotting (n=3). (**C**) HASMCs were treated with PDGF-BB (40 ng/mL) or plus rhFGF21 (0.5 mg/mL) for 18 h in complete medium. The expression of SRF and MYOCD was determined by Western blotting (n=3). (**D**) HASMCs were transfected with HA-FGF21 for 12 h in serum-free medium. Then the cells were treated with PDGF-BB (40 ng/mL) for 18 h in serum-free medium. The expression of SRF, MYOCD and HA was determined by Western blotting (n=3). **(F, G)** HASMCs in a 48-well plate were transfected with pCMV-HA/HA-FGF21 for 24 h. Immunofluorescence assay was used to detect the expression and nuclear location of SRF (**F**) and MYOCD (**G**) (n=3). Data information: Data are expressed as the means ± SDs. Student’s t-test (two groups) or one-way ANOVA followed by Tukey’s test (more than two groups), **P*<0.05.


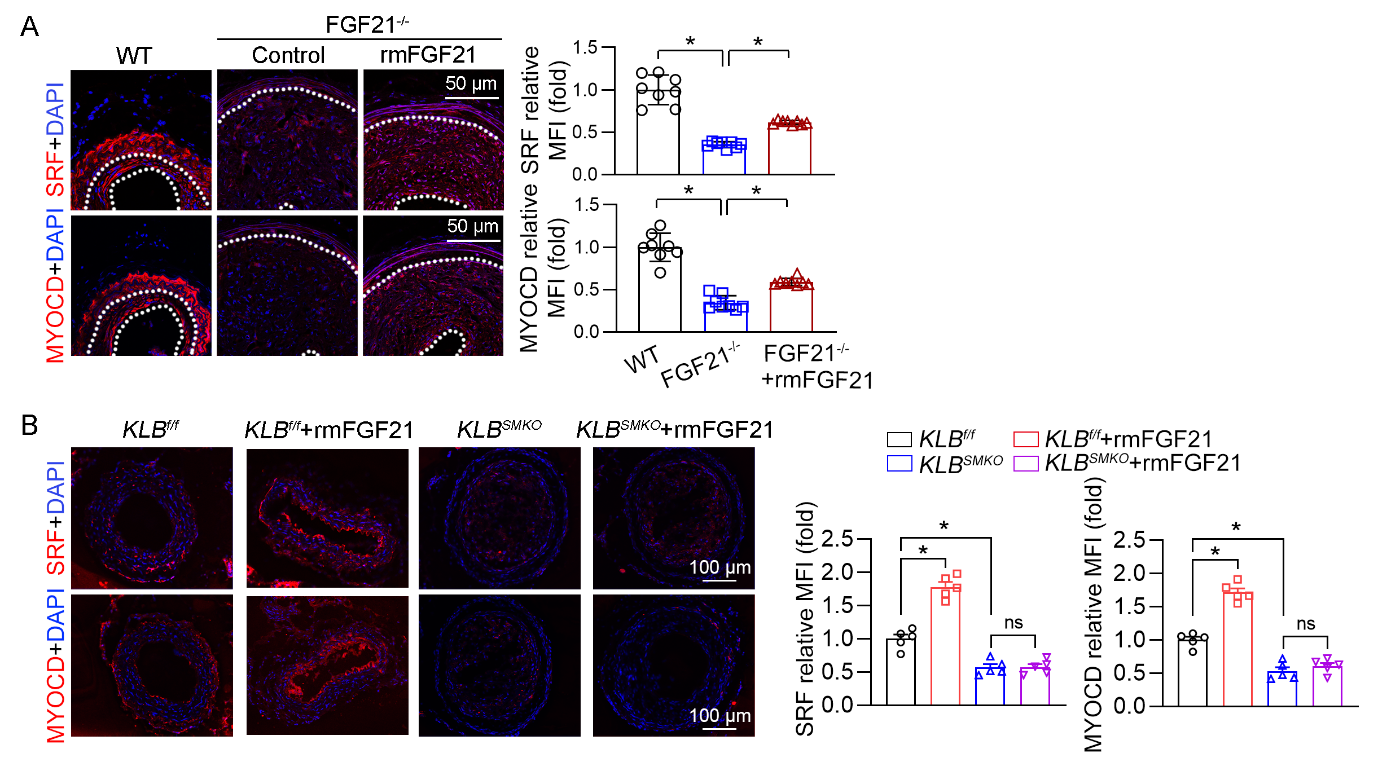


**Figure S5.** **FGF21-increased SRF and MYOCD expression depends on KLB expression *in vivo***

(**A**) Left carotid artery ligation were performed to female C57BL/6J and FGF21^-/-^ mice (n=8), in which FGF21^-/-^ mice was intravenously injected with mouse recombinant protein FGF21 (rmFGF21, 600 μg/kg) twice a week for 4 w. Expression of SRF and MYOCD in neointimal areas was determined by immunofluorescence staining (n=8). (**B**) Left carotid artery ligation were performed to female KLB^f/f^ and KLB^SMKO^ mice (n=5), in which mouse recombinant protein FGF21 (rmFGF21, 600 μg/kg) was intravenously injected twice a week for 4 w. At the end of the experiment, carotid artery samples were individually collected. Expression of SRF and MYOCD in neointimal areas was determined by immunofluorescence staining (n=5). Data information: Data are expressed as the means ± SDs. One-way ANOVA or two-way ANOVA followed by Tukey’s test, **P*<0.05, ns: not significant difference.


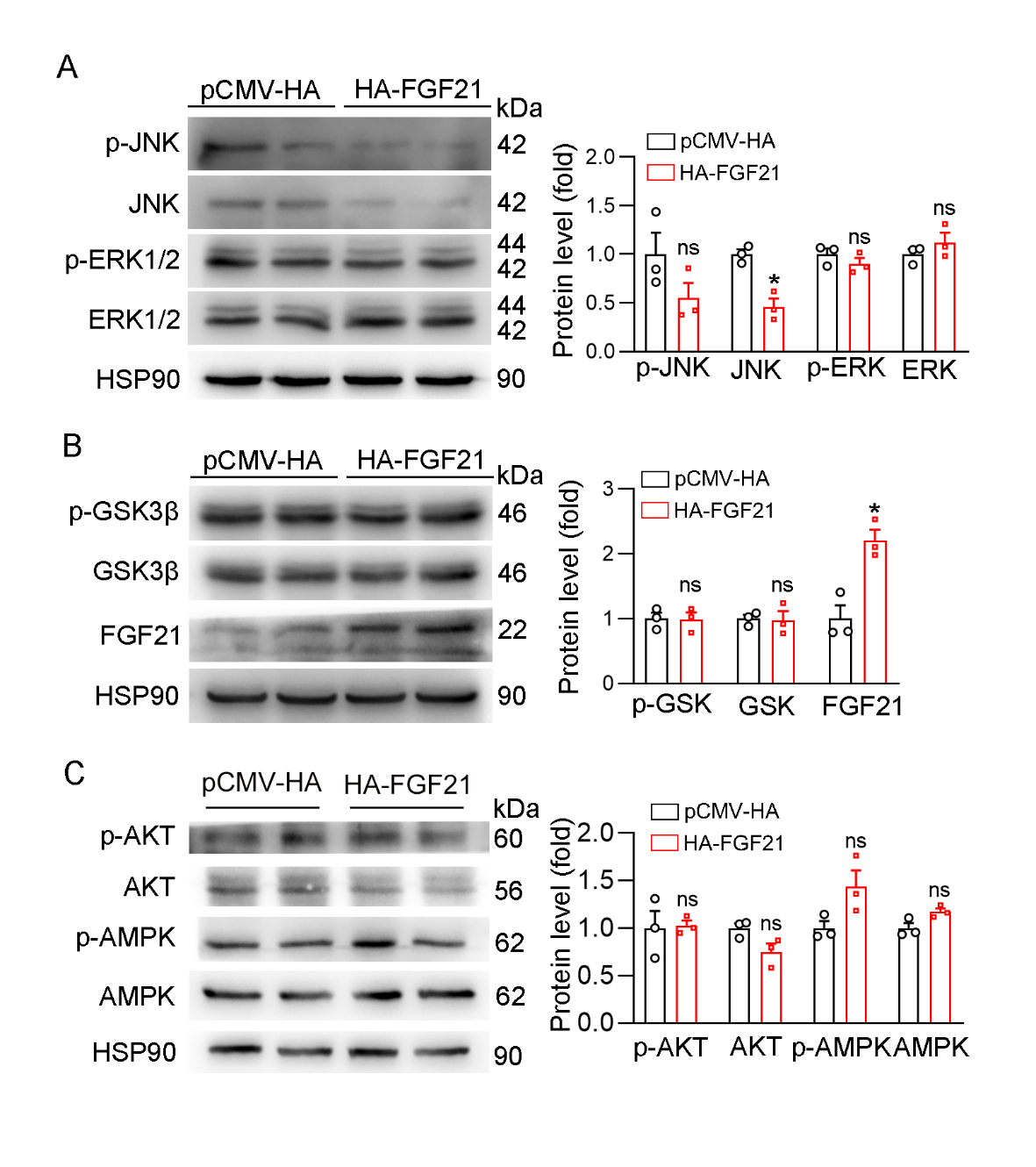


**Figure S6. FGF21 has little effect on JNK, ERK1/2, GSK3β, AKT and AMPK signaling pathways**

HASMCs were transfected with FGF21 overexpression vector for 12 h. After cultured in complete medium for another 24 h, total protein was isolated and used to determine p-JNK, JNK, p-ERK1/2, ERK1/2 (**A**), p-GSK3β, GSK3β, FGF21 (**B**), p-AKT, AKT, p-AMPK and AMPK (**C**) protein expression by Western blotting (n=3). Data information: Data are expressed as the means ± SDs. Student’s t-test, **P*<0.05; ns: not significant difference.


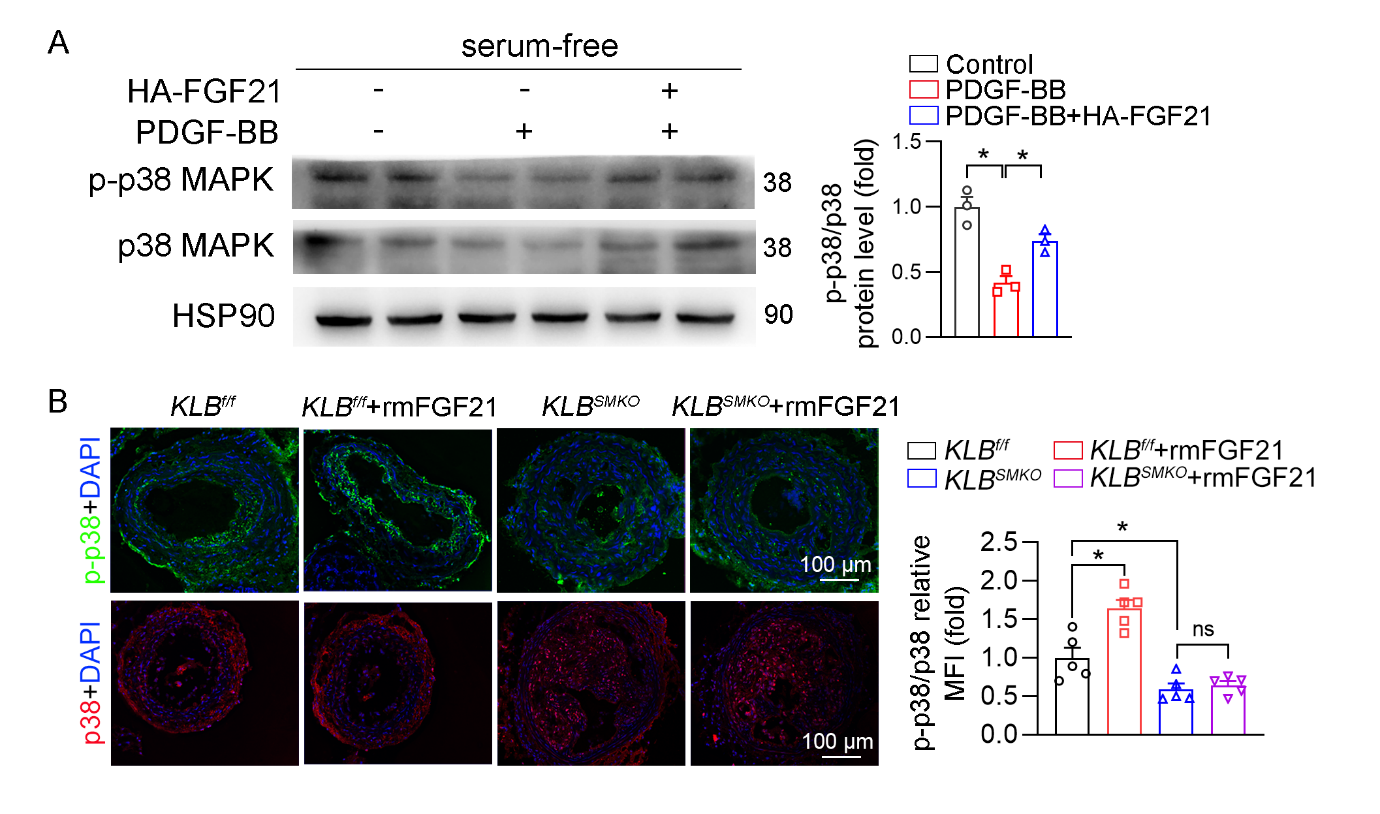


**Figure S7. FGF21 increases p-p38 MAPK expression in a KLB-depended manner**

(**A**) HASMCs were transfected with HA-FGF21 for 12 h in serum-free medium. Then the cells were treated with PDGF-BB (40 ng/mL) for 18 h in serum-free medium. The expression of p-p38 MAPK and p38 MAPK was determined by Western blotting (n=3). (**B**) Female KLB^flox/flox^ and KLB^SMKO^ mice were performed left carotid artery ligation, mouse recombinant protein FGF21 (rmFGF21, 600 μg/kg) was intravenously injected twice a week for 4 w. At the end of the experiment, carotid artery samples were individually collected. The expression of p-p38 MAPK and p38 MAPK in neointimal areas was determined by immunofluorescence staining (n=5). Data information: Data are expressed as the means ± SDs. Student’s t-test (two groups) or two-way ANOVA followed by Tukey’s test (more than two groups), **P*<0.05; ns: not significant difference.


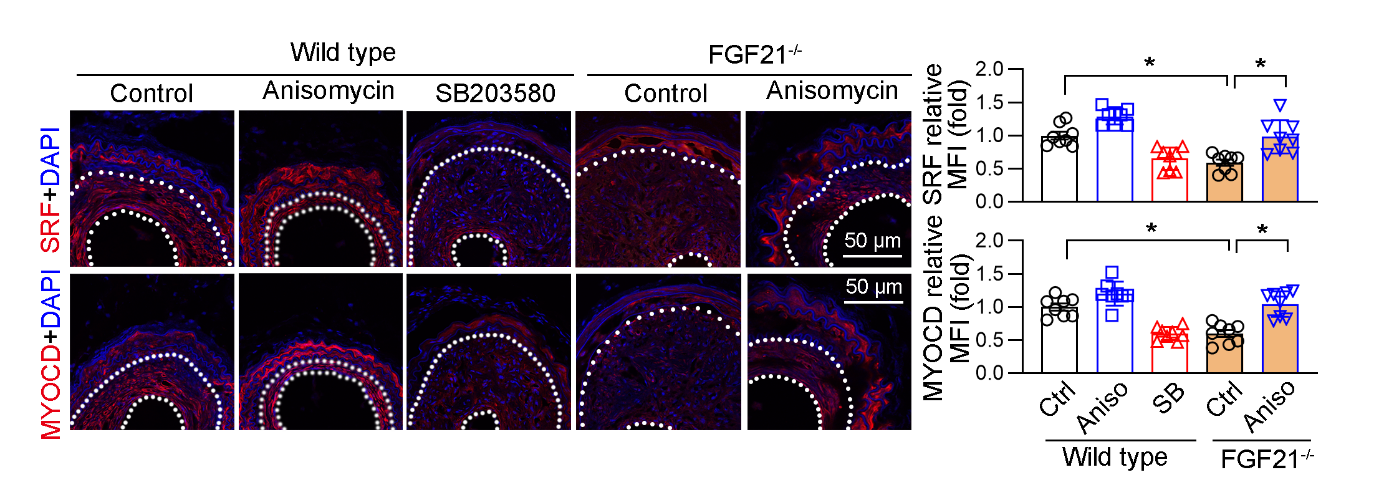


**Figure S8. p38 MAPK activation increases SRF and MYOCD expression**

Female C57BL/6J or FGF21^-/-^ mice were performed left carotid artery ligation, and then intraperitoneally injected with vehicle (distilled water), SB203580 (10 mg/kg) or anisomycin (15 mg/kg) every day, respectively, for 4 weeks. At the end of the experiment, carotid artery samples were collected. The expression of SRF and MYOCD in neointimal areas was determined by immunofluorescence staining (n=8). Data information: Data are expressed as the means ± SDs. Two-way ANOVA followed by Tukey’s test, **P*<0.05.


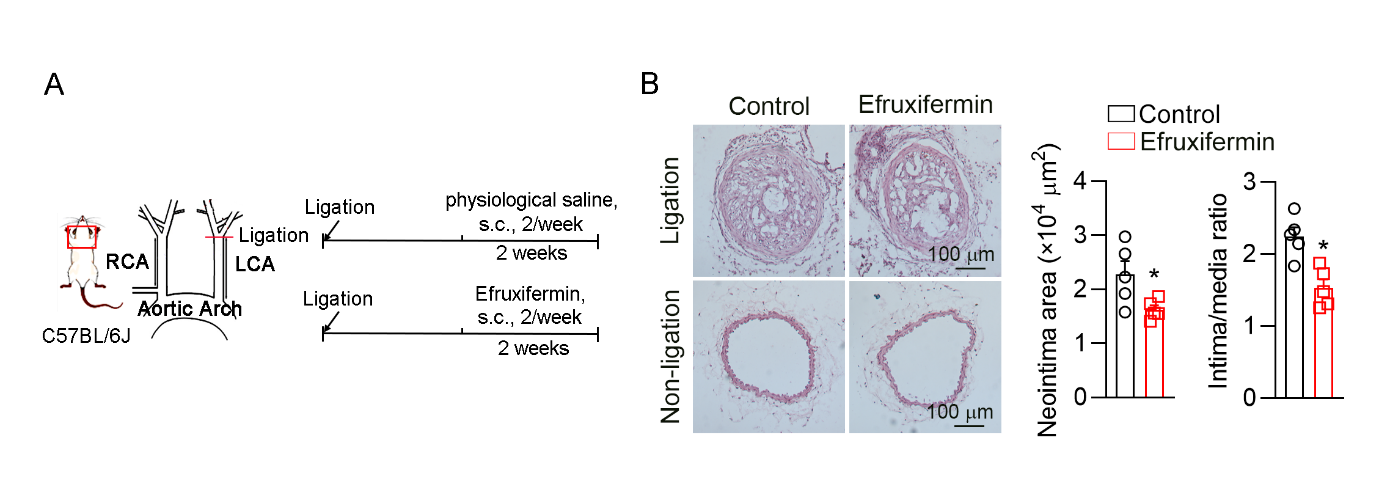


**Figure S9. Efruxifermin treatment reduces established neointima formation**

(**A, B**) Left carotid artery ligation was performed on female C57BL/6J mice (n=5). After two weeks, the mice were injected subcutaneously with physiological saline or efruxifermin (5 mg/kg) once a week for two weeks (**A**). At the end of the experiment, carotid artery samples were individually collected. HE staining for morphological analysis with quantitative analysis of neointima and media areas (**B**). Data information: Data are expressed as the means ± SDs. Student’s t-test, **P*<0.05.

**III. Supplementary Tables**

**Table S1. Antibodies**

| Target antigen | Vendor or Source | Catalog # | Working concentration | Lot # |
| --- | --- | --- | --- | --- |
| SMA | Affinity Biosciences | BF9212 | 1:10000 (WB)  1:200 (IF) | #84o1870 |
| SM22a | Affinity Biosciences | AF9266 | 1:2000 (WB)  1:200 (IF) | #18b5359 |
| SRF | Proteintech | 16821-1-AP | 1:6000 (WB)  1:200 (IF) | 00081771 |
| OPN | Proteintech | 22952-1-AP | 1:4000 (WB)  1:200 (IF) | 00085407 |
| MYOCD | Affinity Biosciences | DF2434 | 1:2000 (WB)  1:500 (IF) | #83t5913 |
| FGF21 | HUABIO | ET1704-04 | 1:5000 (WB) | HK0213 |
| p-p38 MAPK | ABclonal | AP0526 | 1:10000 (WB)  1:200 (IF) | 5500020933 |
| p38 MAPK | ABclonal | A14401 | 1:1000 (WB)  1:200 (IF) | 1159750 |
| KLB | Affinity Biosciences | DF10309 | 1:2000 (WB) | #81k0612/3 |
| FGFR1 | Affinity Biosciences | AF6156 | 1:2000 (WB) | #77u8854 |
| p-FGFR1 | Affinity Biosciences | AF3157 | 1:2000 (WB) | #52s8176 |
| TAK1 | Proteintech | 12330-2-AP | 1:1000 (WB) | 00069297 |
| p-TAK1 | Cell Signaling | #9339 | 1:1000 (WB) | 2 |
| MKK3/6 | Santa Cruz Biotechnology | sc-136982 | 5 mg/mL | #B0818 |
| p-MKK3/6 | Santa Cruz Biotechnology | sc-8407 | 5 mg/mL | #K2017 |
| HSP90 | Proteintech | 13171-1-AP | 1:10000 (WB) | 00121544 |
| a-TUBULIN | Proteintech | HRP-66031 | 1:10000 (WB) | 21000018 |
| Phosphorylated Serine | Bioss | bs-11993R | 1:3000 (WB) | BA06241931 |
| CNN1 | Affinity Biosciences | DF7671 | 1:3000 (WB) | #72h5136 |
| GAPDH | Proteintech | HRP60004 | 1:10000(WB) | 21005148 |
| GSK3b | Cell Signaling | #12456 | 1:1000（WB） | 4 |
| p-GSK3b | Cell Signaling | D85E12 | 1:1000（WB） | 9 |
| CD68 | Affinity Biosciences | #DF7518 | 1:500（IF） | NA |
| MCP-1 | ABclonal | A7277 | 1:100（IF） | NA |
| JNK | ABclonal | A4867 | 1:2000 (WB) | NA |
| p-JNK | ABclonal | AP0631 | 1:5000（WB） | NA |
| p-ERK1/2 | Proteintech | 80031-1-RR | 1:10000（WB） | NA |
| ERK1/2  HA-tag | ABclonal  Proteintech | A4782  51064-2-AP | 1:1000（WB）  1:10000 (WB) | NA  NA |
| p-Thr | Cell Signaling | #9386S | 1:1000 (WB) | NA |

Table S2. Basal clinical characteristics of the study participants

| Clinical characteristics | Non-ASCAD  (n=28) | ASCAD  (n=28) | *P*-value | PCI without restenosis (n=11) | PCI with restenosis (n=11) | *P*-value |
| --- | --- | --- | --- | --- | --- | --- |
| Age (years) | 58.7±6.5 | 59.3±5.1 | 0.6997 | 57.3±9.3 | 65.6±14.9 | 0.1337 |
| Sex M/F | 13/15 | 15/13 | 0.593 | 5/6 | 8/3 | 0.387 |
| Smoking | 9 (32.1) | 12 (42.9) | 0.408 | 6 (54.5) | 3 (27.3) | 0.387 |
| Hypertension | 11 (39.3) | 16 (57.1) | 0.181 | 8 (72.7) | 6 (54.5) | 0.659 |
| Diabetes | 1 (3.6) | 4 (14.3) | 0.349 | 2 (18.2) | 5 (45.5) | 0.361 |
| Plasma TC (mM) | 4.62±0.97 | 4.78±1.40 | 0.6389 | 4.95±0.78 | 3.06±0.28 | <0.0001 |
| Plasma LDL-C (mM) | 2.89±0.69 | 3.16±0.92 | 0.2352 | 3.21±0.59 | 1.37±0.34 | <0.0001 |
| Plasma HDL-C (mM) | 1.16±0.21 | 1.15±0.27 | 0.9263 | 1.11±0.23 | 1.04±0.26 | 0.4697 |
| Plasma TG (mM) | 1.55±0.80 | 1.45±0.89 | 0.6740 | 1.82±0.74 | 1.20±0.65 | 0.0557 |
| EF (%) | 61.00±7.95 | 62.42±7.63 | 0.5310 | 62.27±9.00 | 59.57±29.62 | 0.6482 |
| Coronary lesion length (mm) | / | 9.75±5.77 | / | / | / | / |
| Coronary plaque burden | / | 0.65±0.21 | / | / | / | / |
| Medication |  |  |  |  |  |  |
| Anticoagulants | / | 5 (17.9) | / | 9 (81.8) | 8 (72.7) | >0.999 |
| Statins | / | 10 (35.7) | / | 5 (45.5) | 6 (54.5) | >0.999 |
| ACEI/ARB | 6 (21.4) | 14 (50.0) | 0.026 | 4 (36.4) | 3 (27.3) | >0.999 |
| b-blocker | 4 (14.3) | 6 (21.4) | 0.485 | 2 (18.2) | 3 (27.3) | >0.999 |

TC, total cholesterol; LDL-C, low-density lipoprotein cholesterol; HDL-C, high-density lipoprotein cholesterol; TG, triglyceride; EF, ejection fraction. Data are shown as mean ± SD or n (%). Pearson chi square test was used to compare the gender, smoking, hypertension and diabetes between the non-ASCAD group and the ASCAD group, and Fisher exact test was used to compare the gender, smoking, hypertension and diabetes between the PCI without restenosis group and the PCI with restenosis group. The *P* value for other parameters were calculated by unpaired Student’s T-test.

**Table S3. Sequences of primers for qPCR**

| Gene | Forward primer | Reverse primer |
| --- | --- | --- |
| FGF21(h) | ACTCCAGTCCTCTCCTGCAA | GCACAGGAACCTGGATGTCT |
| SMA(h) | AGAACATGGCATCATCACCA | TACATGGCTGGGACATTGAA |
| SM22a(h) | GGAGCAGTGGGTGCATTTCA | TGCACTAGCCAAGTCATCCG |
| MYH11(h) | GGAGGATGAGATCCTGGTCA | TCTTTAGCCGCACTTCCAGT |
| CNN1(h) | ACTTCATCAAGGCCATCACC | CTCCCACGTTCACCTTGTTT |
| SRF(h) | CACCTACCAGGTGTCGGAGT | GGTGCCAGGTAGTTGGTGAT |
| MYOCD(h) | CAAGCCAAAGGTGAAGAAGC | TAGCTGAATCGGTGTTGCTG |
| FGF21(m) | GTGTCAAAGCCTCTAGGTTTCTT | GGTACACATTGTAACCGTCCTC |
| KLB(m) | TGTTCTGCTGCGAGCTGTTAC | CCGGACTCACGTACTGTTTTT |

**References**

1. Chen Y, Duan Y, Kang Y, Yang X, Jiang M, Zhang L, Li G, Yin Z, Hu W, Dong P, Li X, Hajjar D P, Han J. Activation of liver X receptor induces macrophage interleukin-5 expression. *J Biol Chem*. 2012;287:43340-43350.

2. Zhang B, Zhang Z, Xia S, Xing C, Ci X, Li X, Zhao R, Tian S, Ma G, Zhu Z, Fu L, Dong J T. KLF5 activates microRNA 200 transcription to maintain epithelial characteristics and prevent induced epithelial-mesenchymal transition in epithelial cells. *Mol Cell Biol*. 2013;33:4919-4935.

3. Hu W, Zhang W, Chen Y, Rana U, Teng R J, Duan Y, Liu Z, Zhao B, Foeckler J, Weiler H, Kallinger R E, Thomas M J, Zhang K, Han J, Miao Q R. Nogo-B receptor deficiency increases liver X receptor alpha nuclear translocation and hepatic lipogenesis through an adenosine monophosphate-activated protein kinase alpha-dependent pathway. *Hepatology*. 2016;64:1559-1576.
